# In Search of an Integrative Method to Study Unconscious Processing: An Application of Bayesian and General Recognition Theory Models to the Processing of Hierarchical Patterns in the Absence of Awareness

**DOI:** 10.5334/joc.411

**Published:** 2025-01-06

**Authors:** Antonio Prieto, Pedro R. Montoro, Mikel Jimenez, José Antonio Hinojosa

**Affiliations:** 1 Departamento de Psicología Básica I, UNED, Spain; 2 Department of Psychology, University of Durham, Durham, United Kingdom; 3 Instituto Pluridisciplinar, Universidad Complutense de Madrid, Madrid, Spain; 4 Departamento de Psicología Experimental, Procesos Cognitivos y Logopedia, Universidad Complutense de Madrid, Madrid, Spain; 5 Centro de Investigación Nebrija en Cognición (CINC), Universidad de Nebrija, Madrid, Spain

**Keywords:** Visual awareness, Awareness measures, Unconscious perception, Bayesian inference, Visual masking, General recognition theory

## Abstract

The dissociation between conscious and unconscious perception is one of the most relevant issues in the study of human cognition. While there is evidence suggesting that some stimuli might be unconsciously processed up to its meaning (e.g., high-level stimulus processing), some authors claim that most results on the processing of subliminal stimuli can be explained by a mixture of methodological artefacts and questionable assumptions about what can be considered non-conscious. Particularly, one of the most controversial topics involves the method by which the awareness of the stimuli is assessed. To address this question, we introduced an integrative approach to assess the extent to which masked hierarchical stimuli (i.e., global shapes composed of local elements) can be processed in the absence of awareness. We combined a priming task where participants had to report global or local shapes, with the use of subjective and objective awareness measures collected either in a separate block (offline), or trial-by-trial during the main task (online). The unconscious processing of the masked primes was then evaluated through two different novel model-based methods: a Bayesian and a General Recognition Theory modeling approach. Despite the high correlation between awareness measures, our results show that the use of alternative approaches based on different theoretical assumptions leads to diverging conclusions about the extent of the unconscious processing of the masked primes.

## 1. Introduction

The scientific study of consciousness is not only one of the most relevant and hot topics in the current scientific scene, it is also arguably one of the last frontiers in human knowledge (Block, 2002; Chalmers, 2003; Dennett, 2002; Graziano, 2019, 2022; Jimenez et al., 2024; Lau, 2022). Over the recent years, the field has witnessed an exponential growth in the research volume devoted to it and its applications (Michel et al., 2019), and also the proliferation of a number of theories from different fields such as philosophy, psychology, neurosciences, physics or even mathematics, which have tried to explain the problem of subjective experience (Northoff & Lamme, 2020; Seth & Bayne, 2022). Within consciousness research, and particularly in the field of cognitive science, one of the most controversial and debated issues refers to the study of unconscious processing and the dissociation between conscious and nonconscious perception (Rothkirch et al., 2022; Rothkirch & Hesselmann, 2017; Shanks et al., 2021; Vadillo et al., 2022). The main disagreement stems from the degree to which participants’ behavior may be affected by information that is not consciously accessed (Breitmeyer, 2015; Yaron et al., 2023). Interestingly, the field has been characterized by a pendulum-like swing with respect to the scope of unconscious processing, with some researchers arguing in favor of high-level unconscious processes (Biderman et al., 2020; Mudrik et al., 2014; Soto et al., 2011), while others argue that most of the unconscious processing findings could be explained by some pervasive methodological issues, along with certain widespread erroneous assumptions when interpreting the results (Newell & Shanks, 2014; Shanks et al., 2021; Vadillo et al., 2022).

According to these criticisms, a crucial challenge when inferring the presence of unconscious processing is related to the way in which the awareness of the allegedly unconscious stimuli is measured (Jimenez et al., 2024; Sandberg et al., 2010; Timmermans & Cleeremans, 2015). Although several heterogeneous methods has been proposed to measure the contents of consciousness, they might be divided according to two independent axes, namely the objective-subjective, and the online-offline dimensions (Jimenez et al., 2024; Seth et al., 2008; Timmermans & Cleeremans, 2015). First, awareness measures can be divided into *objective* and *subjective* (Jimenez et al., 2024; Persuh, 2018). Objective or performance-based measures typically involve instructing the participants to discriminate between two different stimuli, usually through a two-alternative forced-choice task (i.e., 2AFC).^1^ Sensitivity (d’) to the task is then taken as a bias free measure (independent of the response criterion) of the awareness of the stimuli: if individuals can discriminate between the two stimuli (d’ > 0), then they must have been aware of them. As the absence of awareness is equated to the absence of performance in the prime discrimination task, unconscious processing is demonstrated by the indirect influence of the unconscious stimuli over subsequent processing (e.g., priming effects). Although objective measures became the “gold standard” in consciousness research until the mid-1980s, they have been criticized on many different grounds. The first is the absence of exclusiveness, in other words, the d’ = 0 criterion may rule out also unconscious perception. The second also stems from the d’ = 0 criterion, as it means to assume that the absence of evidence for any conscious processing is taken as evidence of absence (Timmermans & Cleeremans, 2015). The third, and perhaps most obvious, is that objective measures do not actually estimate conscious experience, but only task performance (Lau, 2007), which also means that, in principle, any device that performs above chance in the discrimination task (e.g., a photodiode) would be consciously perceiving the stimuli (Persuh, 2018). On the other hand, subjective or experience-based measures rely on the introspective reports of the individuals, which are directly related to their phenomenal experiences (Overgaard et al., 2010; Persuh, 2018; Sandberg et al., 2010). Specifically, different types of subjective measures have been proposed in consciousness research, from the visibility (either dichotomous or graded) reports, including the PAS scale employed in the present study, to different metacognitive measures (e.g., confidence ratings and post-decision wagering; see Jimenez et al., 2024 for a thorough review on the topic of measuring conscious experience). When assessing awareness in this way, participants are usually asked to report their conscious perception of the stimulus on a dichotomous, graded, or even a continuous scale (Pournaghdali et al., 2023; Ramsøy & Overgaard, 2004; Sergent & Dehaene, 2004). This procedure is thought to be a more direct approach to the study of (un)conscious perception, as the evidence for the existence of unconscious processing is obtained directly from the “no visibility” reports of the participants. However, subjective measures have also been questioned on different fronts. Major criticisms involve their susceptibility to response biases (Eriksen, 1960; Merikle et al., 2001) and criterion confounds^2^ (Bachmann, 2015; Kahneman, 1968). Particularly, shifts to a more conservative or liberal response criterion could bias participants’ reports of awareness, even within the same experiment when different tasks are employed (Jimenez et al., 2020, 2023).

Second, we can also operatively classify awareness measures into the *online* and *offline* dimension, according to the point in time at which they were obtained (Jimenez et al., 2024; Kiefer et al., 2023). Offline (e.g., retrospective) measures of awareness include any type of measurement that is taken once an ongoing task has been completed. Studies following this approach typically ask participants to the assess stimulus awareness in a separate block following the main task. Therefore, offline measures (whether objective or subjective) do not interfere with task performance (e.g., the priming task in a dissociation paradigm). However, their retrospective nature raises the concern of “retrospective assessment” (Shanks & John, 1994) or the “immediacy problem” (Newell & Shanks, 2014), which highlight the need to asses consciousness concurrently, or as soon as possible after the behavior. In addition, offline measures have faced further criticism due to their lack of sensitivity to intra-individual fluctuations in visual awareness over trials (Bengson & Hutchison, 2007; Lähteenmäki et al., 2015). Alternatively, online (e.g., concurrent) measures involve assessing participants’ awareness on a trial-by-trial basis during the task at hand, typically (although not always) through subjective measures like the PAS scale (e.g., Jimenez et al., 2023; Kiefer et al., 2023; Sabary et al., 2020). The introduction of online measures allows to examine potential intra-individual fluctuations during the task, and meeting the immediacy criterion proposed by Newell and Shanks (2014). However, online measures are not free from some problems, especially in those tasks in which reaction times are collected (e.g., a priming paradigm) since they become dual-task paradigms. As a consequence, the need to allocate attentional resources to two different tasks slowdown the responses to the main task and increases the variability of the reaction times (Jimenez et al., 2023; Kiefer et al., 2023). The reliability and sensitivity of the assessment of stimulus awareness is also affected (Newell & Shanks, 2014), as online measures usually induce a more conservative awareness criterion bias due to the higher attentional demands of the dual-task design (Jimenez et al., 2023).

Regardless of the different measures and points in time in which the awareness of a stimulus can be assessed, another major issue in the field of consciousness research involves the way in which the different measures of awareness are analyzed and interpreted within a given paradigm. This has led to a wide variety of analysis strategies and criteria for differentiating between conscious and unconscious processing (Jimenez et al., 2024; Timmermans & Cleeremans, 2015). In one of the most widely adopted approach, the classical dissociation paradigm, the strategy consists of analyzing the differences between an indirect performance measure (e.g., priming measure) and an awareness measure (Reingold & Merikle, 1988). Typically, this kind of strategy has been associated with objective and offline measures of awareness (i.e., a forced-choice discrimination task aimed to provide an objective index of stimulus visibility). If the awareness measure indicates chance level discrimination of the stimuli, then we can conclude that any effect found in the indirect performance measure is caused by unconscious processing (Jimenez et al., 2023; Sabary et al., 2020). The main theoretical assumption behind this paradigm is that the sensitivity measure in the discrimination task (usually d’) is an index of the stimulus level of awareness. The problems associated with this approach include the acceptance of this theoretical a priori, along with the issues related to the use of an offline-objective measure of awareness, especially the possibility that the effects found may be due to the influence of a few conscious trials, even when the average performance in the awareness measure is at chance level (d’ = 0). To overcome this problem, some studies have made use of post hoc data selection (see Rothkirch et al., 2022 for a review) in which participants who are above a specific cut-off (typically chance-level in the objective awareness task) are removed from the data, and only the remaining subset of “unaware” participants enter the data analysis. Then, any effect found in this subset of participants is taken as supporting unconscious perception. However, this procedure has been discouraged as it produces a *regression to the mean*,^3^ which introduces bias as the measurement error is not randomly sampled within the sub-group (Rothkirch et al., 2022; Shanks, 2017). One possible alternative way to overcome this problem is the use of regression methods, in which there is no need of selecting a non-conscious sub-group of participants (in fact, they deliberately establish conditions that allow a proportion of subjects to perform above chance in the awareness measure). Particularly, Greenwald et al. (1995) proposed a regression-based method to predict unconscious effects from objective awareness measures (e.g., d’), in a linear regression model where the regression intercept represents the predicted effect for an ideal observer whose awareness in the objective forced-choice measure is equal to zero. Unfortunately, although the regression method proposed by Greenwald et al. (1995) avoids the post-hoc selection of participants, it does not solve the source of the problem.^4^ As it was the case with the post-hoc selection of a subgroup of participants, the presence of measurement error in the predictor variable (the awareness measure) leads to underestimating the slope coefficient. This is a well-established issue known as regression attenuation or dilution (a phenomenon equivalent from a psychometric point of view to the regression to the mean discussed above), that usually involves the overestimation of the regression intercept. Indeed, further studies showed that this method overestimates unconscious effects due to the underestimation of the regression slope (Dosher, 1998; Sand & Nilsson, 2016). In response to this problem, some authors have proposed different solutions to deal with the problem of measurement error and regression attenuation (Klauer et al., 1998; Matzke et al., 2017). Specifically, Goldstein et al. (2022) recently developed a Bayesian generative model solution for estimating the intercept of the regression model while accounting for the measurement error. The authors build upon a Bayesian general method to correct for measurement error in the estimation of correlations developed by Matzke et al. (2017), and adapt their model to the particular case of unconscious processing, in order to estimate the intercept in a regression model while accounting for error in the awareness and the effect measures.

Alternatively, and also within the priming paradigm, other studies have introduced online-subjective reports to concurrently measure participants’ awareness (Sabary et al., 2020; Van den Bussche et al., 2013). The trial-by-trial awareness assessment allows to analyze conscious and unconscious trials separately, thus meeting the immediacy criterion proposed by Newell and Shanks (2014), but they are subjected to all of the criterion problems commented earlier, including biases and shifts within and between subjects and tasks (see Jimenez et al., 2020 for a review). Importantly, irrespective of the methodological issues, the use of subjective measures within the priming paradigm introduces a change of theoretical assumptions in unconscious processing research. From employing task accuracy as a measure of awareness to the use of subjective phenomenological reports to identify unconscious trials, thus shifting from equating awareness to performance, to linking consciousness with the sensitivity to a feature that *feels like something* for the participant. The question therefore arises as to the extent to which both are comparable, or even whether the objective and subjective thresholds of awareness measure the same underlying construct (Jimenez et al., 2023; Kiefer et al., 2023; Sandberg et al., 2010; Song et al., 2015; Szczepanowski & Pessoa, 2007).

Outside the dissociation paradigm the question has become even more complicated. Usually, these studies involve a masked detection/discrimination and/or categorization task in which awareness is evaluated following one of the different methods described above. For example, subjective-offline measures are employed in studies in which participants are required to discriminate or categorize masked stimuli and, after completing the task, are asked to indicate (via subjective report) whether they saw the stimuli (Juruena et al., 2010; Killgore & Yurgelun-Todd, 2004). Others have employed subjective-online measures, assessing the awareness of the stimulus after each trial either through dichotomous or graded judgments, and first order (visibility) or second order (metacognitive) reports (Jimenez et al., 2018, 2021; Pournaghdali et al., 2023; Sandberg & Overgaard, 2015; Song et al., 2015). Interestingly, in many of these works the interpretation of the sensitivity measures (usually d’) is reversed, from being a measure of the degree of awareness (therefore assuming the necessity of a d’ = 0 to conclude the presence of unconscious processing) to being a measure of performance in the -allegedly unconscious- task, thus assuming that a d’ = 0 indicates the absence of processing of the stimuli whether conscious or unconscious. From this last perspective, Pournaghdali et al. (2023) conducted a recent study to analyze the relationship between perceptual processing and awareness in which they collected online-subjective awareness measures while participants had to discriminate between the emotional expression of masked face stimuli. To analyze the existence of unconscious processing of the emotional expressions Pournaghdali and colleagues employed a novel method based on the General Recognition Theory (GRT), a multivariate extension of the signal detection theory (SDT), adapted to situations in which stimuli vary in more than one dimension, in this case emotional valence and awareness (see also Ashby & Soto, 2015 for a detailed revision of the GRT). Fitting this model allowed the authors to construct sensitivity vs awareness (SvA) curves that specify the sensitivity (d’) for emotional expression discrimination as a function of the relative likelihood of awareness (RLNA). Thus, in this approach the presence of some level of sensitivity (d’ > 0) when the RLNA is high is an indicative of unconscious processing. Once again, and beyond the methodological issues described, the question is whether these diverse paradigms and measures to study non-conscious processing are assessing the same phenomena, or at least if the produce comparable results across tasks and measures, a question that has been previously raised by some authors (Jimenez et al., 2023, 2024; Kiefer et al., 2023; Michel, 2023; Szczepanowski & Pessoa, 2007).

To address these questions, in the present study we aim to explore unconscious visual processing through an integrative paradigm that includes objective and subjective measures of awareness collected both during the main task (online) and in separate blocks (offline). In addition, we will examine unconscious processing through the different classical analysis strategies associated with various combinations of awareness measures and research paradigms (e.g., dissociation paradigm, trial-wise post-hoc selection), together with some new approaches recently proposed by different authors and based on models derived from Bayesian analysis and General recognition theory (Goldstein et al., 2022; Pournaghdali et al., 2023).

To this end, we will focus on the unconscious processing of non-hierarchical patterns formed by the perceptual grouping of individual stimuli into a global form, and to what extent the local elements and the global shape formed by their arrangement can occur outside the observer’s awareness (Jimenez et al., 2017, 2023; Koivisto & Revonsuo, 2004; Sabary et al., 2020). The question of which processes are involved in perceptual organization has been extensively debated for decades (Peterson & Kimchi, 2013; Wagemans et al., 2012a; Wagemans et al., 2012b), but the interplay between perceptual organization and visual awareness is still not well understood. Classical cognitive theories assume that perceptual grouping (as part of the perceptual organization process) can occur preattentively in the absence of attention (Julesz, 1981; Neisser, 1967; Treisman, 1985), and at least some types of perceptual grouping along with other perceptual organization phenomena (e.g., contour integration, illusory contour completion) have been found in subliminal processing conditions (Jimenez & Montoro, 2018; Jimenez et al., 2017; Lamy et al., 2006; Montoro et al., 2014; Russell & Driver, 2005). However, the current evidence seems to depend on: (1) the perceptual organizational processes under study, something that seems reasonable given that perceptual organization comprises a multiplicity of processes with different time courses, developmental trajectories and attentional demands (Kimchi, 2009; Sabary et al., 2020); and (2) the methods employed to prevent the stimulus from entering consciousness (Moors et al., 2016). Indeed, previous evidence on the integration of local elements into global shapes in the absence of awareness already exists (Jimenez et al., 2023; Sabary et al., 2020) and, crucially for our interest, this evidence derives from a variety of awareness measures and different analysis strategies. For example, Sabary et al. (2020) explored the integration of local elements into a global shape in the absence of awareness by combining a masked priming paradigm and online-subjective measures, in which subjective awareness reports were obtained in a trial-by-trial basis using the Perceptual Awareness Scale (PAS) developed by Ramsøy and Overgaard (2004). The authors concluded that the local elements were represented in the absence of visual awareness, but conscious processing was necessary for grouping them into a global shape. On the contrary, in a recent study, Jimenez et al. (2023) expanded these findings by introducing two different masked priming paradigms in which objective (offline) and subjective (PAS scale, both online and offline) awareness measures were collected. Interestingly, the authors implemented a Bayesian regression model previously employed by Goldstein et al. (2022) to predict the priming effect for ideal observers whose awareness is at chance level, thus trying to overcome the limitations of the post-hoc selection of participants and trials, typical of studies that use objective and subjective measures of awareness respectively. Interestingly, the combined results of these two studies showed local unconscious priming effects when assessed through subjective-online measures of awareness (Sabary et al., 2020), and the unconscious integration of local elements into a global shape when measured through objective-offline measures of awareness and analyzed employing Bayesian regression models.

However, even though these two studies and, especially, the work by Jimenez et al. (2023) is an example of the integration of different measures combined with multiple task conditions, still suffer from the absence of some specific awareness measures and analysis strategies. Specifically, the authors did not collect objective online measures of awareness, nor did they analyzed the unconscious processing of the primes directly, beyond the indirect measures derived from the masked priming (dissociation) approach. Also, in the case of Jimenez et al. (2023) they did not investigate local priming effects. Therefore, in the present work we expand the paradigm devised by Jimenez et al. (2023) by including objective awareness measures in a trial-by-trial manner (online), along with a new model-based analysis of the association between the subjective measures of awareness and the perceptual processing of the masked primes, based on a multidimensional version of the signal detection theory (GRT). Moreover, both local and global priming effects were analyzed. Particularly, we conducted four experiments focused on the role of awareness in processing the local and global structure of hierarchical patterns, employing a classical masked priming design with hierarchical stimuli similar to the ones employed by Jimenez et al., (2023) and Sabary et al., (2020), to explore the unconscious processing of local and global primes using three alternative approaches: the classical dissociation paradigm, and the two newly proposed Bayesian and General Recognition Theory models. We implemented an exhaustive design in which objective and subjective, as well as offline and online measures of awareness were combined, allowing us a thorough comparison of the prime sensitivities (d’) and the indirect priming effects for the different combinations of awareness measures. In the first two experiments, global shapes (squares or diamonds) made of neutral (i.e., non-related to de global forma) local elements (triangles and circles) were presented as primes in a masked priming paradigm. In the two remaining experiments, an array of local elements (squares or diamonds) forming a neutral (i.e., non-related to the identity of the local elements) global shape (triangles and circles) were presented as primes in the same masked priming paradigm. All four experiments included three different blocks, each one associated to a different task: (1) a single-task priming block where participants gave speeded responses to the probe identity (which could be congruent or incongruent with the prime) while ignoring the primes; (2) a multiple-task priming block in which besides responding to the probes, participants were asked to indicate the identity of the prime (objective-online awareness measure) and to give a visibility report using the PAS scale (subjective-online awareness measure), so participants had to attend to the prime and the probe simultaneously within the same trial; and (3) a visibility block where participants ignored the probe and the objective and subjective awareness measures of the prime were collected offline (separate from the priming task). Note that the inclusion of three different tasks also entails three distinct levels of attention to the prime, which allowed us to explore how attention modulates the way in which the primes were processed, a relevant issue considering the ongoing debate between proponents of theories of consciousness that equate attention to consciousness (Dehaene et al., 2006), and those who dissociate both processes (Koch & Tsuchiya, 2007; Lamme, 2003). In addition, as the prime duration and specially the SOA between the prime and the backward mask has proven to be a relevant factor in constructing the global shape under no awareness conditions (Jimenez et al., 2017, 2023; Sabary et al., 2020), we introduced two different SOAs (40 and 53 ms) in each priming condition (global/local) that produced divergent results in previous studies. Finally, we also incorporated two minor but relevant improvements over the previous studies already discussed (Jimenez et al., 2023; Sabary et al., 2020). First, to improve the implementation of the GRT-based model, we doubled the number of catch trials to 64 (20% of the total number of trials). Second, we matched the stimuli presentation conditions across all blocks by including the probe stimulus also in the visibility block, even though participants were not required to respond to it.

According to the results of previous works, we expected slower RTs in the multiple-task compared to the single-task condition, due to the need to divide the attention between the primes and the probes in the former. Crucially, we expected this attentional split to increase the variability of the RTs and affect the facilitation (congruency) effects in the priming task, thus eliminating or at least attenuating any possible unconscious priming effects in the multiple-task compared to the single-task condition. Regarding the prime/mask SOA, we expected an increase in the priming effects as the SOA increases, in line with previous studies showing that the total time that the prime is available for its perceptual processing is a crucial factor in determining unconscious effects. Finally, we also expected a higher level of unconscious processing of primes during the visibility block, due to the higher level of attention paid to the primes compared to the multiple-block.

Regarding the visibility of the primes during the different blocks, we anticipated higher values in the awareness measures during the visibility block, compared to the multiple-task block, given the higher level of attention allocated to the primes during that block.

There is mixed evidence about the extent to which objective and subjective measures of awareness assess the same underlying process, with some studies pointing to the existence of a clear dissociation (Szczepanowski & Pessoa, 2007), whereas others showed a high degree of convergence between them (Jimenez et al., 2023; Kiefer et al., 2023). Here, we expected a high correlation between objective and subjective measures within the same block, and between the same measure across different blocks, considering that previous studies employing the same paradigm have found significant overlaps between the two different measures.

## 2. Methods

### 2.1. Participants

All participants were recruited among the population of undergraduate Psychology students at UNED (all aged between 19 and 62). All reported normal or corrected-to-normal vision and received course credit as reward for their participation. Sample size was calculated according to a mixed sample stopping rule (Jimenez et al., 2023; Rouder, 2014; Schönbrodt et al., 2017) based on both a minimum of participants and the cumulative evidence in favor or against the null hypothesis.^5^ Once the minimum number of participants (n = 20) per experiment had been reached, the final number of participants in each experiment was calculated by monitoring the Bayes Factor (BF) values obtained in the Bayesian regression analyses (Goldstein et al., 2022) at the end of each experimental day (Matzke et al., 2015). Particularly, the data collection was stopped once we obtained “substantial” evidence favoring either the null model (regression intercept = 0, BF_01_ ≤ 1/3) or the alternative model (regression intercept ≠ 0, BF_10_ ≥ 3), according to the classification scheme for the Bayes factor developed by Jeffreys (1998). All the experiments were conducted after obtaining the written consent of each participant, were in accordance with the Declaration of Helsinki, and accepted by the UNED and UCM ethics committee.

### 2.2 Apparatus

All the stimuli were displayed in a 19-in. LCD–LED Samsung 943N color monitor with a 75-Hz refresh rate, a 5:4 aspect ratio, and a resolution of 1280 × 1024. The experiments were controlled by a computer running E-Prime 3.0 software (Psychology Software Tools, 1996–2018). Viewing distance was kept constant at approximately 57 cm. Participants performed their responses by means of a keyboard and a mouse connected to the computer. All experiments were conducted in soundproofed experimental cabins under controlled lighting and temperature conditions.

### 2.3 Stimuli

**Figure 1:**
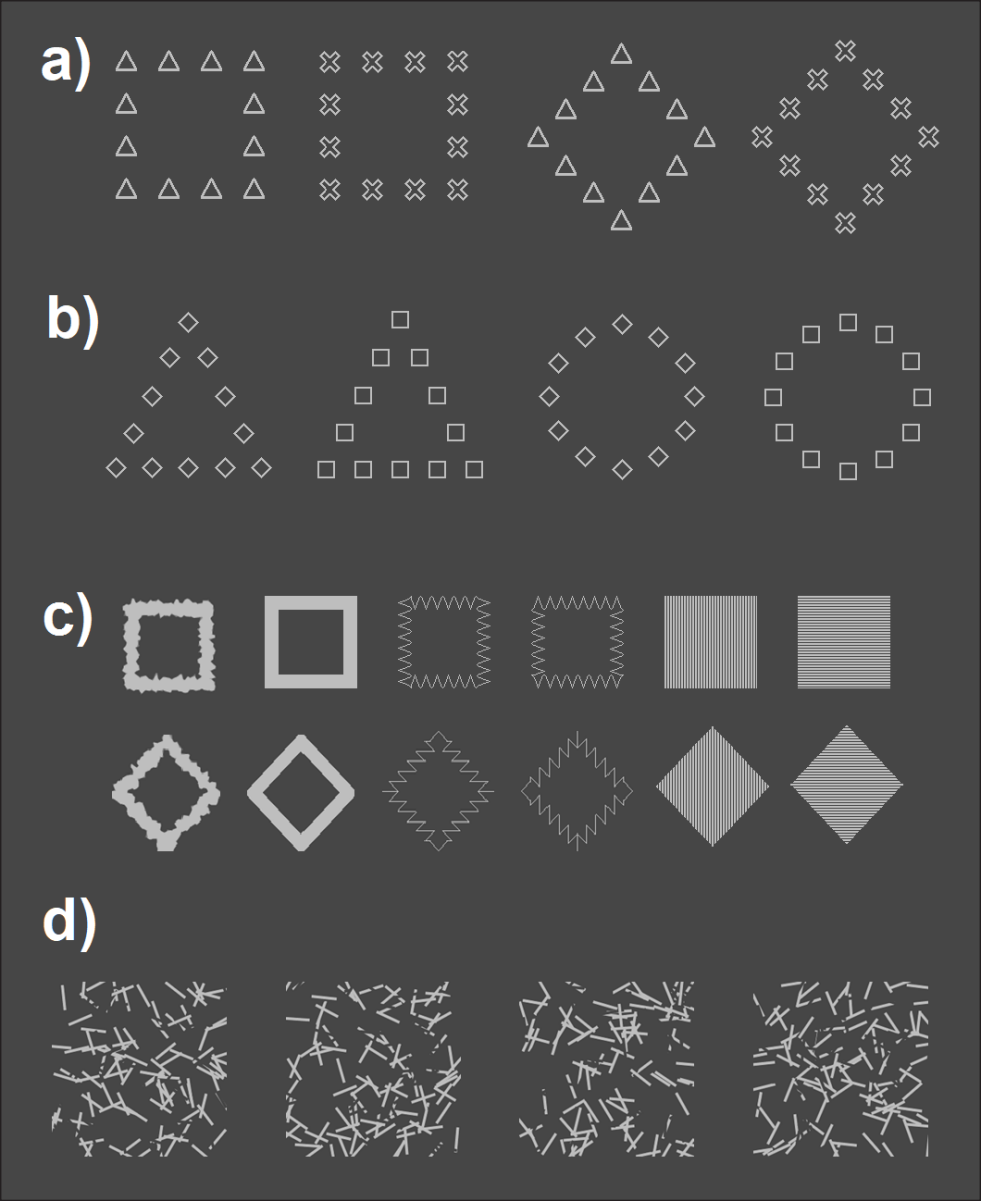
Stimuli used in the four experiments. **(a)** Prime stimuli for global priming experiments. **(b)** Prime stimuli for local priming experiments. **(c)** Probe stimuli for all the experiments. **(d)** Examples of mask stimuli.

The stimuli were displayed at the center of the screen against a gray background (RGB: 70, 70, 70; 3.3 cd/m^2^). All the experiments included four different primes. In the global priming condition, the primes consisted of two global squares or diamonds, made up of twelve local x-cross or triangles. In the local priming condition, the primes consisted of two global circles or triangles made up of twelve diamonds or squares. The side of the global diamond subtended 6°, the side of the global square subtended 5°, the diameter of the global circle subtended5.5°, and the side of the global triangle subtended 5.5°. Each local element, either a square, a diamond, a triangle, or a x-cross subtended 1.7° × 1.7°. All the contours of the local elements were 3 pixels wide (see Figure 1a and 1b). The probes consisted of twelve square or diamond-shaped stimuli with different visual features (see Figure 1c). All the probes were light gray (RGB: 190, 190, 190; 33 cd/m^2^) and smaller than the probes to avoid retinotopic effects. Square probes subtended 3° × 3°, and diamond probes subtended 4° × 4°.

The forward and backward mask were the same employed previously by Sabary et al. (2020) and Jimenez et al. (2023). They consisted of 100 randomly placed light gray lines (RGB: 190, 190, 190; 33 cd/m^2^) against a dark gray (RGB: 70, 70, 70; 3.3 cd/m^2^) background color subtending a 7.5° × 7.5° visual area. Background colored lines were superimposed over light gray lines to create random noise patterns (i.e., line cuts). Each line subtended 1°–1.5° visual angle in length and 4 pixels in width. All the line orientations were randomly sampled, excluding 45° and its multiples to avoid any potential response facilitation by the local elements of the mask (see Figure 1d). A pool of 50 masks was created and randomly presented (only once per trial) during the experiment.

### 2.4. Design and procedure

**Figure 2:**
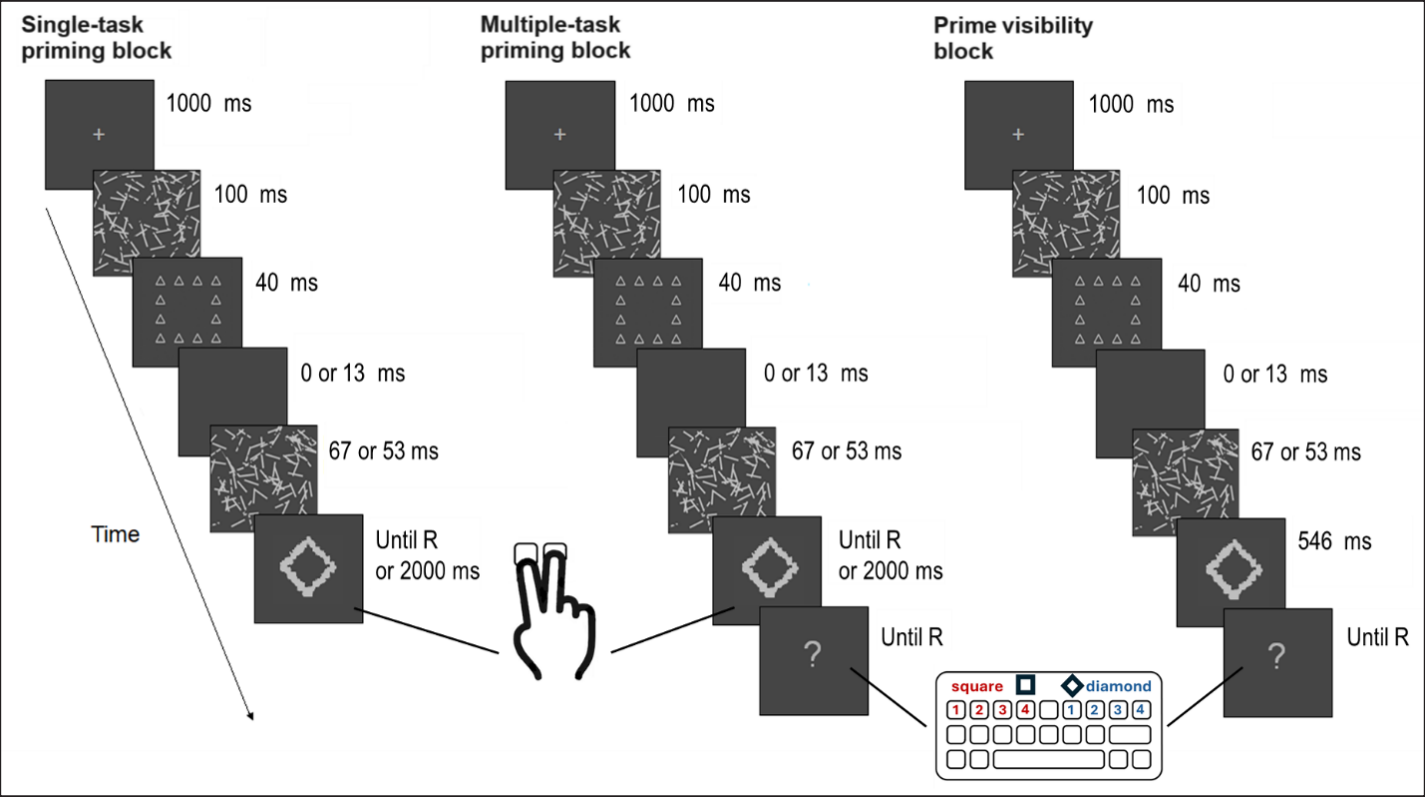
Sequence of events in each block of the experiments.

Four different and independent samples of participants took part in this study (one for each experiment). Each of the four experiments consisted of three different blocks in which participants performed three different tasks (one per block): (1) a single-task priming block, a multiple-task priming block, and a prime visibility block. In Experiments 1 and 2 (global priming condition) a global diamond or square shape made of neutral (circle or triangle) elements was presented as prime stimuli. In experiments 3 and 4 (local priming condition) a global (circle or triangle) neutral shape made of squares or diamond elements was presented as prime stimuli (see Figures 1 and 2). The participants that took part in each of the experiments performed the three blocks sequentially with a short break in between, during which the experimenters gave the participants the instructions for the next block. The experimental session started either with the single-task priming block or the multiple-task priming block, which were counterbalanced across participants within each experiment. Last, after both priming blocks, the participants performed the visibility task block to avoid any possible underestimation of the prime awareness due to increases in prime visibility throughout the experiment (Timmermans & Cleeremans, 2015). In the single and multiple-task priming blocks, each trial started with a fixation cross (1° × 1° light grey cross: RGB 210, 210, 210; 49 cd/m^2^) displayed at the center of the screen for 1000 ms. Then, a forward mask was presented for 100 ms, and followed by a prime that remained in the screen for 40 ms. In Experiments 1 and 3 (global and local priming conditions respectively), a backward mask pattern was presented for 67 ms immediately after the disappearance of the prime. In Experiments 2 and 4 (also global and local priming conditions respectively), a blank inter stimulus (ISI) screen was displayed for 13 ms after the prime. Afterwards, the backward mask pattern was presented for 53 ms, in order to maintain a constant 107 ms prime-target SOA across experiments. Finally, the probe remained on the screen until response or at least 2000 ms. In all experiments, during blocks one and two (single and multiple tasks counterbalanced), participants were required to discriminate the shape of the probe presented (square or diamond) as fast as possible but without making too many errors, by pressing the buttons 1 and 2 of the numeric keypad on the right side of the keyboard with the index and middle finger of the right hand. During the task, the index and middle fingers of the right hand were in constant contact with the corresponding keys on the numeric keypad to ensure that the participant provided speed responses to the probe stimuli. Before the beginning of the single-task priming block, the participants were informed of the presence of the masked primes but were told to ignore them as they were task irrelevant. After the participants consigned their response to the probe, an intertrial blank screen appeared for 800 ms before the beginning of the next trial. In the multiple-task priming block, the trial sequence was identical to the single-task priming block except that, following the response to the probe discrimination task, a question mark was displayed on the screen signaling the participants to provide both a subjective and an objective report of prime visibility. Both subjective and objective judgments were collected simultaneously, by means of two different PAS scales ranging from 1 to 4 (1 = No perception, 2 = Weak perception, 3 = Almost Clear perception and 4 = Clear perception) mapped to the left (*Q, W, E, R* keys) and right (*U, I, O, P* keys) sides of the keyboard. To perform their prime visibility responses, participants were instructed to use the left PAS scale to indicate the visibility of the prime if they think it was a square, and to use the right PAS scale whenever they think the prime presented was a diamond. Therefore, both the objective judgment (shape discrimination) and the subjective judgment (visibility) were collected simultaneously with a single keystroke without time pressure using the left hand, to avoid any possible interference between the speeded probe discrimination task, and the prime shape discrimination and visibility tasks. Participants were explicitly informed that the four categories of the PAS scale referred exclusively to the perception of the global shape of the prime in the global priming condition (Experiments 1 and 2), and to the perception of the local elements in the local priming condition (Experiments 3 and 4), to ensure that participants only informed about the awareness of the relevant dimension/content in each experiment. The specific instructions regarding the Global PAS (Experiments 1 and 2) were: (1) ‘No perception’: There is no subjective experience of the stimulus; (2) ‘Weak perception’: A feeling that some global figure has been shown although this could not be identified, (3) ‘Almost clear perception’: Global shape perceived with some degree of uncertainty. A feeling of almost being certain about one’s answer; (4) ‘Clear experience’: An experience of clearly seeing the global shape. Complete certainty about one’s answer. The specific instructions regarding the local PAS (Experiments 3 and 4) were: (1) ‘No perception’: There is no subjective experience of the stimulus; (2) ‘Weak perception’: A feeling that some local elements have been shown although they could not be identified, (3) ‘Almost clear perception’: local elements perceived with some degree of uncertainty. A feeling of almost being certain about one’s answer; (4) ‘Clear experience’: An experience of clearly seeing the local elements. Complete certainty about one’s answer.

In the prime visibility block participants first performed a forced-choice prime shape discrimination task, followed by a subjective report of the visibility of the prime on the PAS scale. In this block the sequence of events was identical to single-task priming block except that, in this block, after the offset of the second mask, two response options were displayed (“square” and “diamond) on the center of the screen until the participants provide their objective and subjective reports of the visibility of the prime, following the same method described for the multiple-task block (two different scales PAS scales ranging from 1 to 4 mapped to the left and right sides of the keyboard. In this block, participants were explicitly instructed to ignore the probes and focus only on the masked primes, trying to identify them as accurately as possible. They were also told to guess on trials in which they could not identify the primes (and to give a PAS rating of 1 in those cases). Trials were self-administered to ensure that participants were as ready to detect the primes as possible (See Table 1 for an overview of the sequence of events in each block).

**Table 1:** Priming condition and variable display durations (ISI and SOA) in ms across experiments.


EXPERIMENT	PRIMING CONDITION	VARIABLE DISPLAY DURATIONS		PRIME-TARGET SOA

		ISI	BACKWARD MASK	

1	Global	0	67	107

2	Global	13	53	107

3	Local	0	67	107

4	Local	13	53	107


Each block consisted of 320 trials, 20% (64 trials) were catch trials in which no prime was presented on the screen. Before the start of the experimental phase, participants performed 24 practice trials (single and multiple-task priming blocks), or 12 practice trials (prime visibility block). Feedback was provided during the practice trials in the single and multiple-task priming blocks, but not in the prime visibility block. Two breaks within each block (after trials 110 and 220) and two more between blocks (after block 1 and 2) were introduced to avoid fatigue effects. The breaks between blocks were also used to brief the participants with instructions for the next phase. The whole experimental session lasted about 90 minutes (including breaks within and between blocks).

### 2.5. Data analysis

Before the data analysis, incorrect trials (only for RT analyses), and trials with RTs below 200 ms and above 2000 ms, and those above and below 2 standard deviations (*SD*) from the mean of each participant (calculated after removing trials above 200 and below 2000 ms) were removed. In addition, participants with accuracy levels lower than 75% and/or more than 10% “clear perception” subjective reports (PAS = 4) for catch trials in either the multiple-task priming block or the prime visibility block were also excluded from the analyses. All the analyses reported were conducted independently for each of the four experiments, so all variables manipulated were within-subjects’ factors. The Bayesian generative modeling correction to the Greenwald regression method (Goldstein et al., 2022; Greenwald et al., 1995), and the General Recognition Theory (GRT) model-based analysis of the association between awareness and perceptual processing (Ashby & Soto, 2015; Pournaghdali et al., 2023), were performed employing Rstudio (version 2023.09.1 + 494, based on the R programming language version 4.3.2) using the *rjags* (Plummer et al., 2006) and a modified version of the *grtools* (Soto et al., 2017) packages respectively. All the remaining analyses were performed using JASP statistical software (version 0.18.3, www.jasp-stats.org).^6^

The hypothesis that local elements can be grouped together into global shapes (experiments 1 and 2), and that the local elements themselves can produce a reliable priming effect (experiments 3 and 4) in the absence of visual awareness, was tested separately for the single-task priming block, the multiple-task priming block, and the prime visibility block, following complementary approaches. In addition, to compare the results (RTs) from the single-task and the multiple-task blocks we performed complementary analysis (2 × 2 Bayesian repeated-measures ANOVA, with block: single-task vs multiple-task, and congruency: congruent vs incongruent as within-subject factors) that can be found in the supplementary materials. In the case of the single-task priming block, data were analysed by means of two different methods: (1) the classical dissociation paradigm (Reingold & Merikle, 1988); and (2) a Bayesian generative modelling correction to the Greenwald regression method (Goldstein et al., 2022). First RT data were sent to a Bayesian paired samples t-test comparing congruent vs incongruent trials. Bayes factor (BF) provided evidence in favour or against the existence of a priming effect by the masked primes. To estimate the degree to which the participants were able to discriminate the masked primes above chance-level (and, hence, the level of awareness of the primes), prime shape discrimination responses from the prime visibility task were transformed into an objective sensibility measure (d’_obj_) of prime visibility. Once calculated, Bayesian one sample t-tests were conducted to assess the degree to which there is evidence to support chance-level (d’ = 0) prime shape discrimination.

In addition, priming effects in the single-task priming block were also analyzed by means of a Bayesian generative modelling correction to the Greenwald regression method (Goldstein et al., 2022; Greenwald et al., 1995). In this method, priming scores are predicted from centered awareness scores,^7^ with the regression intercept representing the predicted effect for a given participant whose performance (proportion of correct responses) in the prime shape discrimination task is at chance level (and, therefore, is completely unaware of the primes). To prevent the overestimation of the intercept due to the regression attenuation (see Behseta et al., 2009 and Goldstein et al., 2022 for a complete description), we corrected the Greenwald regression procedure employing the Bayesian generative modeling approach developed by (Goldstein et al., 2022), which accounts for measurement error and corrects the bias (overestimation) of the intercept estimation by approximating a posterior distribution for the intercept along with a 95% credible confidence interval, and calculating a BF comparing the evidence for a null model (intercept = 0, absence of subliminal priming effects) against the evidence in favor of a model in which the intercept ≠ 0 (presence of subliminal priming effects).^8^

The analysis of the multiple-task priming block data also followed a combined approach. First, RTs from trials in which participants gave PAS-1 reports were sent to a Bayesian paired samples t-test comparing congruent vs incongruent conditions.^9^ Second, RT data (unfiltered) were sent to a Bayesian paired samples t-test comparing congruent vs incongruent trials following the same procedure outlined for the single-task priming block. In this case, the objective sensibility measure (d’_obj_) of prime visibility necessary to assess the degree of awareness of the primes during the multiple-task priming block, was calculated based on the responses to the prime shape discrimination task performed during this block (see 2.4 section). Once d’_obj_ was calculated, Bayesian one sample t-tests were conducted (in the same way described for the simple-task priming block) to assess the degree to which there is evidence to support chance-level (d’ = 0) prime shape discrimination. Third, priming effects in the multiple-task priming block were analyzed by means of the same Bayesian generative modelling correction to the Greenwald regression described for the single-task priming block.

To further deepen into the unconscious processing of the primes, data from the objective prime shape discrimination task, and prime subjective visibility judgment using the PAS scale in both the multiple-task and the visibility block, were subjected to a general recognition theory (GRT) model-based analysis of the association between awareness and the perceptual processing of the primes (Pournaghdali et al., 2023). This model was then employed to build sensitivity vs awareness (SvA) curves to characterize the relationship between the perceptual processing of the primes (sensitivity to discriminate the shape of the primes) and the level of awareness.^10^ Specifically, the data from the prime visibility block were fitted to a two-dimensional GRT model (GRT with individual differences, or GRT-wIND; Ashby & Soto, 2015; Pournaghdali et al., 2023). To ensure that the selected model was the best fit for the data without overfitting, we followed the procedure previously employed by Pournaghdali et al. (2023) and fitted 16 versions of the model using maximum likelihood estimation.^11^ Then we conducted the model selection using the Akaike information criterion (AIC) and chose the best model among the candidates. Once the best GRT model was selected, two values were computed in order to construct the SvA curves: (1) the likelihood that no stimulus was presented over the likelihood that a stimulus was presented, given a particular value in the awareness distribution (i.e., the relative likelihood of no awareness or RLNA), and (2) the conditional sensitivity (d’_cond_), or the d’ computed from the unidimensional normal distribution of square and diamond primes, conditional on a value of the awareness dimension. 95% confidence intervals were obtained by simulating 1,000 data samples from the best GRT model obtained in the previous step. This resulted in a distribution function for SvA curves, and the limits for the confidence intervals were taken from the percentiles of this function. Once the SvA curves and their respective confidence intervals were constructed, they allowed us to evaluate the dependence of the visual processing of the primes from awareness.

Finally, to investigate to what extent both objective and subjective prime awareness measures obtained during the multiple-task block (online measures) and the prime visibility block (offline measures) converge, we compared them by transforming both to a common sensitivity measure (d’) following and adapting the procedure depicted by Szczepanowski and Pessoa (2007) and previously used by Jimenez et al. (2023). In this method, sensitivity for the objective measures of prime visibility (d’_obj_) was calculated for each participant by treating one level (e.g., square primes) as the signal and the other level (e.g., diamond primes) as noise during the objective prime shape discrimination tasks performed in the multiple-task priming block and the prime visibility block. On the other hand, d’ for the subjective measures (d’_subj_) was calculated by assuming that trials in which the prime was presented, and participants indicated being aware of it (PAS = 2, 3 and 4) correspond to “hits”, whereas trials in which the prime was not presented (catch trials) and participants indicated being aware of its presentation were considered “false alarms”. This allowed us to compare prime awareness estimations derived from objective (d’_obj_) and subjective (d’_subj_) measures within a common signal detection theory (SDT) framework, as well as to analyze possible differences between the objective and subjective judgments given in the multiple-task priming block and the prime visibility block due to the different requirements of the tasks. To analyze to what extent objective and subjective measures differ within the same task, and the possible effect of the different tasks on the sensitivity measures, a 2 × 2 repeated measures Bayesian ANOVA with block (multiple-task priming block, prime visibility block) and d’-type (objective vs subjective) as within-subjects factors was conducted. Bayesian Model Averaging (Wagenmakers et al., 2018; Haldane 1932; Hoeting, Madigan, Raftery, & Volinsky, 1999) was computed to compare models that contain a specific effect to equivalent models without the effect. In addition, we performed Bayesian correlations between sensitivity measures to analyze the degree to which d’_obj_ and d’_subj_ were related within the same task and whether a given sensitivity measure was consistent across the different blocks, particularly we were interested in the correlation between the same measure across different blocks, and the correlation between different measures within the same block.

## 3. Results

All the tests conducted below are available at the Open Science Repository https://doi.org/10.17605/OSF.IO/G3AKU (see also Supplementary Materials).

### 3.1 Experiment 1: global priming SOA 40 ms

Twenty-three participants took part in Experiment 1: 16 women, age range = 43 (M = 30.64, SD = 11.93). The number of participants that took part in the experiment was calculated by monitoring the Bayes factor (BF) in the regression analysis (Goldstein et al., 2022), following a mixed Bayesian stopping rule (see section 2.1 for a detailed description). Two participants were replaced due to their low accuracy levels (<75%) in the multiple-task block. Four participants were replaced due to their incorrect use of the PAS scale (>10% of “clear perception” reports in the catch trials in either the multiple-task or visibility blocks). Participants’ accuracy in the probe discrimination task was very high (single-task; Congruent: *M* = .97, *SD* = 0.14, Incongruent: *M* = .97, *SD* = 0.16; multiple-task; Congruent: *M* = .97, *SD* = 0.16, Incongruent: *M* = .96, *SD* = 0.19), so only RTs data were analyzed.

#### Single-task block analyses

**Figure 3:**
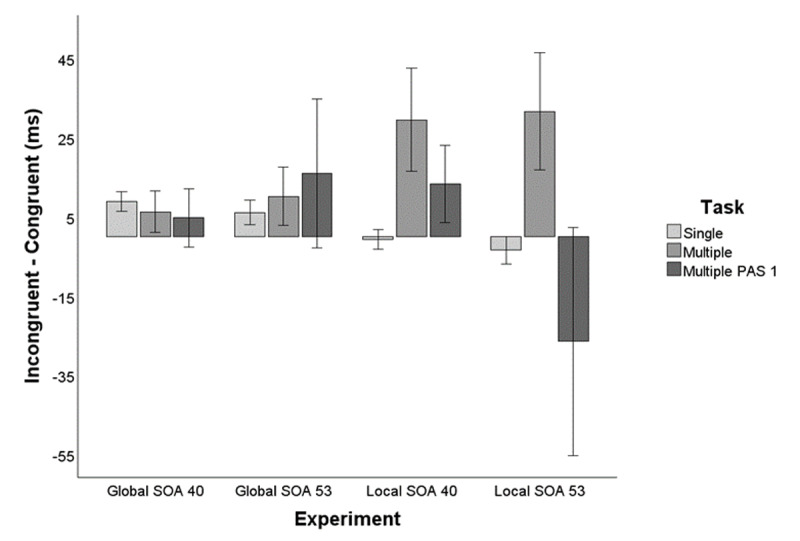
Priming effects (Incongruent – Congruent) in both the single-task priming block, the dual-task priming block (unfiltered), and the dual-task priming block (filtered by PAS1). Error bars represent standard error (SE) of the mean.

First, and following the classical dissociation paradigm (Reingold & Merikle, 1988) we performed a Bayesian paired samples t-test on the individual mean RTs comparing congruent and incongruent trials. The analysis showed strong evidence (BF_10_ = 39.336, error % = 4.5 × 10^–4^) favoring the existence of a priming effect by the global shape. The RTs obtained in the single task (Congruent: *M* = 517 ms, *SE* = 9.9 ms; Incongruent: *M* = 526 ms, *SE* = 10.4 ms) were 39.95 times more likely under the alternative hypothesis (i.e., faster RTs in the congruent relative to incongruent trials). See Figure 3 and Table 2.

**Table 2:** Mean (M) and standard error (SE) for RTs (ms) in congruent and incongruent conditions in the single-task priming block, multiple-task priming block (unfiltered), and the multiple-task priming block (filtered by PAS1) across all four experiments.


TASK	RT			

	CONGRUENT		INCONGRUENT	
	
	M	SE	M	SE

Single				

Global 40 ms	518	9.9	527	10.4

Global 53 ms	548	14.3	554	12.7

Local 40 ms	520	13.3	519	13.6

Local 53 ms	569	20.9	565	20

Multiple				

Global 40 ms	780	42.4	786	43.5

Global 53 ms	859	46.3	870	46.4

Local 40 ms	858	46.2	888	53

Local 53 ms	951	53	983	60.3

Multiple PAS-1				

Global 40 ms	765	40	770	40.7

Global 53 ms	852	46.1	877	49.9

Local 40 ms	814	41.15	828	42.73

Local 53 ms	957	64.1	889	53.15


To analyze the extent to which participants were unaware of the primes according to an objective measure of discriminability, responses to the prime shape discrimination task during the visibility block (*M* = .55, *SE* = 0.023) were transformed into a d’_obj_ sensibility measure (*M* = 0.32, *SE* = 0.142) (Green & Swets, 1966; Tanner & Swets, 1954). Mean d’_obj_ scores were then sent to a Bayesian one sample t-test. Results provided moderate evidence (BF_10_ = 3.451, error % = 7.435 × 10^–5^) favoring group visibility being above chance level. Data were 3.45 times more likely under the alternative hypothesis (d’_obj_ > 0) than under the null hypothesis (d’_obj_ = 0).

**Figure 4:**
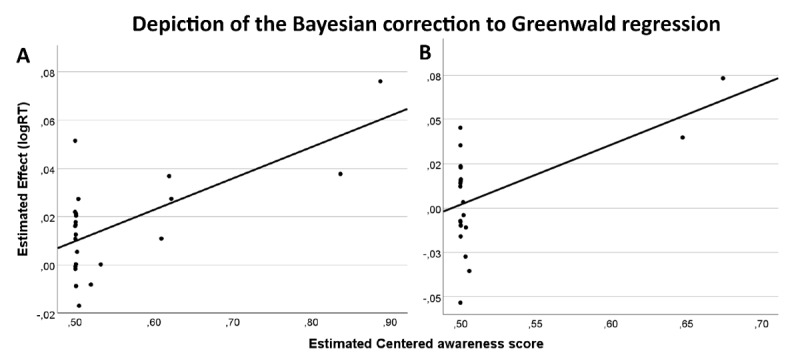
Depiction of the Bayesian correction to Greenwald regression in Experiment 1 (Global SOA 40) in the single-task **(A)** and multiple-task **(B)** blocks. Each circle represents the participant estimated true score. The x-axis represents the centered awareness score, the y-axis represents the estimated effect (incongruent – congruent conditions). The intercept is the expected performance for completely unaware participants.

Last, RT data from the single task were also analyzed employing the Bayesian generative modelling correction to the Greenwald regression method (Greenwald et al., 1995) developed by Goldstein et al. (2022). After fitting the regression model, we found moderate evidence (BF_10_ = 0.282 C.I = 7.928 × 10^–5^/0.019) against a priming effect by the global shape, indicating that the data were 3.54 times more likely under the null model in which the intercept = 0 (no subliminal priming effect) compared to a model drawn from a wide normal distribution (see Figure 4A).

#### *Multiple-task* block *analyses*

During the multiple-task priming block participants performed three different tasks: (1) a probe discrimination task already performed in the single-task block; (2) a prime shape discrimination task; and (3) a prime visibility judgment using the PAS scale (see section 2.4 for a detailed description). PAS reports distribution was as follows: PAS-1 (no perception of the prime) was reported on 70,5% of the trials, PAS-2 (weak perception) on 19,8% of the trials, PAS-3 (almost clear perception) on 8,4% of the trials, and PAS-4 (clear perception) on 1,4% of the trials (see Table 3).

**Table 3:** Mean (M) and standard error (SE) for PAS reports (%) in the multiple-task priming block and the prime visibility block across all four experiments.


PAS	EXPERIMENT							

	GLOBAL 40 MS		GLOBAL 53 MS		LOCAL 40 MS		LOCAL 53 MS	
			
BLOCK	M	SE	M	SE	M	SE	M	SE

Multiple-task								

1	70.5	6	65	6.9	52.7	6.3	51.4	8.4

2	19.8	4	22	4.6	21.7	3	19	4

3	8.4	2.6	7.6	2.8	14.8	3.3	12.6	3.4

4	1.4	1.1	5.4	3.7	10.8	3.7	17	5.6

Prime Visibility								

1	64.1	6.5	52.3	7.6	17.2	5.1	18.8	5.6

2	18.7	3.4	25.2	4.1	20.9	3.6	22.2	5.4

3	11.2	2.7	16.3	4.4	24.9	4.2	22.1	4.7

4	6	2.9	6.2	3.3	37	7.5	36.9	7.5


First, and given that in the multiple-task block participants performed both the probe discrimination task and the prime shape discrimination task, we first analyzed RT data following the classical dissociation paradigm (Reingold & Merikle, 1988) already employed for the single task. We performed a Bayesian paired samples t-test on the individual mean RTs comparing congruent and incongruent conditions in all trials. The analysis showed anecdotical evidence (BF_10_ = 0.716, error % = 0.007) favoring the absence of a priming effect by the global shape. The RTs obtained in the multiple task (Congruent: *M* = 780 ms, *SE* = 42.36 ms; Incongruent: *M* = 786 ms, *SE* = 43.48 ms) were 1.39 times more likely under the null hypothesis (i.e., no differences in RTs between congruent and incongruent conditions). See Figure 3 and Table 2.

Responses to the prime shape discrimination task during the multiple-task block (*M* = .51, *SE* = 0.01) were transformed into a d’_obj_ sensibility measure (*M* = 0.073, *SE* = 0.060). Mean d’_obj_ scores were then sent to a Bayesian one sample t-test. Results provided anecdotical evidence (BF_10_ = 0.721, error % = 0.010) favoring group visibility being at chance level. Data were 1.386 times more likely under the null hypothesis (d’_obj_ = 0) than under the alternative hypothesis (d’_obj_ > 0)

Second, the individual mean RT data for PAS-1 reports (no perception of the primes) were sent to a Bayesian paired samples t-test comparing congruent and incongruent trials. The results showed moderate evidence (BF_10_ = 0.380, error % = 3.835 × 10^–6^) against the existence of a congruency effect produced by the global shape in the priming task during the multiple-task block (PAS1-Congruent: *M* = 765 ms, *SE* = 40.01; PAS1-Incongruent: *M* = 770 ms, *SE* = 40.68). See Tables 2 and 3 and Figure 3.

Multiple-task RT data were also analyzed employing the Bayesian generative modelling correction to the Greenwald regression method developed by Goldstein et al. (2022). During the multiple-task block participants performed a prime shape discrimination task, therefore, we used the data from this task as the awareness score to predict priming scores in the absence of awareness. The results showed moderate evidence (BF_10_ = 0.110, C.I –0.027/0.092) against a priming effect by the global shape. Data were 9.09 times more likely under the null model (intercept = 0, no subliminal priming effect) compared to a model drawn from a wide normal distribution (see Figure 4B).

### 3.2 Experiment 2: global priming SOA 53 ms

Twenty participants took part in Experiment 2: 16 women, age range = 39 (M = 30.95, SD = 12). Six participants were replaced due to their incorrect use of the PAS scale (>10% of “clear perception” reports in the catch trials in either the multiple-task or visibility blocks). Participants’ accuracy in the probe discrimination task was very high (single-task; Congruent: *M* = .98, *SD* = 0.04, Incongruent: *M* = .98, *SD* = 0.05; multiple-task; Congruent: *M* = .97, *SD* = 0.03, Incongruent: *M* = .97, *SD* = 0.02), so only RTs data were analyzed.

#### Single-task block analyses

Bayesian paired samples t-test on the individual mean RTs comparing congruent and incongruent trials showed anecdotal evidence (BF_10_ = 2.254, error % = 7.170 × 10^–5^) favoring the existence of a priming effect by the global shape. The RTs obtained in the single task (Congruent: *M* = 548 ms, *SE* = 14.29; Incongruent: *M* = 554 ms, *SE* = 12.72 ms) were 2.25 times more likely under the alternative hypothesis (faster RTs in the congruent relative to incongruent trials). See Figure 3 and Table 2.

Bayesian one sample t-test performed on the transformed responses (d’_obj_, *M* = 0.52, *SE* = 0.212) to the prime shape discrimination task during the visibility block (*M* = .59, *SE* = 0.034), provided moderate evidence (BF_10_ = 4.684, error % = 5.462 × 10^–5^) favoring group visibility being above chance level. Data were 4.68 times more likely under the alternative hypothesis (d’_obj_ > 0) than under the null hypothesis (d’_obj_ = 0)

**Figure 5:**
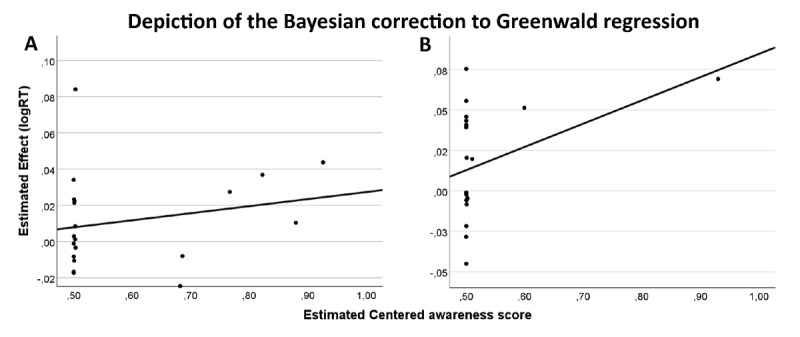
Depiction of the Bayesian correction to Greenwald regression in Experiment 2 (Global SOA 53) in the single-task **(A)** and multiple-task **(B)** blocks. Each circle represents the participant estimated true score. The x-axis represents the centered awareness score, the y-axis represents the estimated effect (incongruent – congruent conditions). The intercept is the expected performance for completely unaware participants.

Last, after fitting the Bayesian generative modelling correction to the Greenwald regression, we found moderate evidence (BF_10_ = 0.251, C.I = –0.001/0.024) against a priming effect by the global shape, indicating that the data were 3.98 times more likely under the null model in which the intercept = 0 (no subliminal priming effect) compared to a model drawn from a wide normal distribution (see Figure 5A).

#### Multiple-task block analyses

During the multiple-task block PAS reports distribution was as follows: PAS-1 was reported on 65% of the trials, PAS-2 on 22% of the trials, PAS-3 on 7.6% of the trials, and PAS-4 on 5.4% of the trials (see Table 3).

The Bayesian paired samples t-test on the individual mean RTs comparing congruent and incongruent conditions in all trials showed moderate evidence (BF_10_ = 0.109, error % = 3.005 × 10^–4^) favoring the absence of a priming effect by the global shape. The RTs obtained in the multiple task (Congruent: *M* = 859 ms, *SE* = 46.27; Incongruent: *M* = 870 ms, *SE* = 46.40) were 9.17 times more likely under the null hypothesis (i.e., no differences in RTs between congruent and incongruent conditions). See Figure 3 and Table 2.

Bayesian one sample t-test performed on the transformed responses (d’_obj_, *M* = 0.23, *SE* = 0.16) to the prime shape discrimination task during the visibility block (*M* = .53, *SE* = 0.02) provided anecdotal evidence (BF_10_ = 1.054, error % = 0.010) favoring group visibility not being at chance level. Data were 1.05 times more likely under the alternative hypothesis (d’_obj_ > 0) than under the null hypothesis (d’_obj_ = 0).

Individual mean RT data for PAS-1 reports were sent to a Bayesian paired samples t-test comparing congruent and incongruent trials. The results showed anecdotal evidence (BF_10_ = 0.510, error % = 1.620 × 10^–6^) against the existence of a congruency (priming) effect produced by the global shape in the priming task during the multiple-task block (PAS1-Congruent: *M* = 852 ms, *SE* = 46.13; PAS1-Incongruent: *M* = 877 ms, *SE* = 49.92). See Table 2 and Figure 3.

Last, the results from the Bayesian generative modelling correction to the Greenwald regression showed moderate evidence (BF_10_ = 0.1538053, C.I –0.039954/0.028277) against a priming effect by the global shape. Data were 6.5 times more likely under the null model (intercept = 0, no subliminal priming effect) compared to a model drawn from a wide normal distribution (see Figure 5B).

### 3.3 Experiment 3: local priming SOA 40 ms

Twenty-six participants took part in Experiment 3 (24 women, age range = 38 years; M = 28.57; SD = 11.51). Two participants were replaced due to an incorrect use of the PAS scale (i.e., more than 10% “clear perception” reports for catch trials in either the multiple-task or the prime visibility blocks). Participants responses were extremely accurate (single-task; Congruent: *M* = .99, *SD* = 0.09, Incongruent: *M* = .97, *SD* = 0.13; dual-task; Congruent: *M* = .98, *SD* = 0.12, Incongruent: *M* = .98, *SD* = 0.12), so only RT data were analyzed.

#### Single-task block analyses

A Bayesian paired samples t-test was performed comparing individual mean RTs for congruent vs incongruent trials. The results showed moderate evidence (BF_10_ = 0.166, error % = 9.982 × 10^–5^) against a priming effect by the local elements (Congruent: *M* = 520 ms, *SE* = 13.29; Incongruent: *M* = 519 ms, *SE* = 13.59; see Table 2 and Figure 3).

Prime detection responses from the prime visibility task (*M* = .83, *SE* = 0.03) were transformed into a sensibility measure (d’_obj;_ *M* = 2.551, *SE* = 0.31; see Table 3) and sent to a Bayesian one sample t-test. The results (BF_10_ = 1.546 × 10^6^, error % = 3.652 × 10^–9^) indicated very strong evidence favoring group visibility of the primes being above chance level (d’_obj_ > 0).

**Figure 6:**
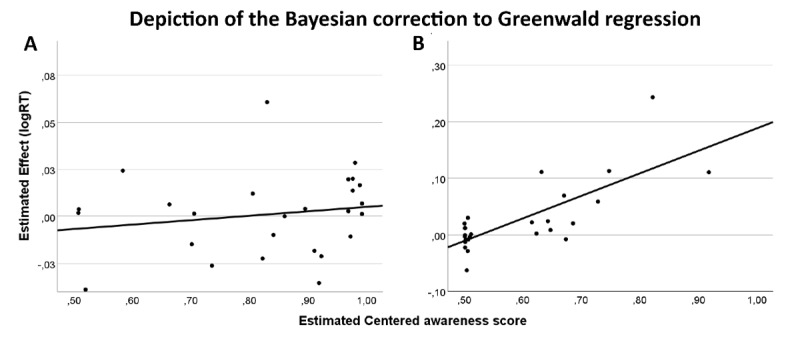
Depiction of the Bayesian correction to Greenwald regression in Experiment 3 (Local SOA 40) in the single-task **(A)** and multiple-task **(B)** blocks. Each circle represents the participant estimated true score. The x-axis represents the centered awareness score, the y-axis represents the estimated effect (incongruent – congruent conditions). The intercept is the expected performance for completely unaware participants.

Last, the results from the Bayesian generative regression model showed strong evidence (BF_10_ = 0.086, C.I –0.023/0.013) against a priming effect by the local elements. Data were 11.66 times more likely under the null hypothesis where the intercept of the regression model is set to 0 (no subliminal priming effect), compared to the alternative hypothesis (indicating a subliminal effect) (see Figure 6A).

#### Multiple-task block analyses

The multiple-task priming block distribution of PAS reports was as follows: PAS1 (no perception of the prime) was reported on 52.7% of the trials, PAS2 (weak perception) on 21.7% of the trials, PAS3 (almost clear perception) on 14.8% of the trials, and PAS4 (clear perception) on 10.8% of the trials (see Table 3).

The results from the Bayesian paired samples t-test on the individual mean RTs comparing congruent and incongruent conditions in all trials indicated moderate evidence (BF_10_ = 3.557, error % = 8.812 × 10^–5^) favoring the existence of a priming effect by the local elements. The RTs obtained in the multiple task (Congruent: *M* = 858 ms, *SE* = 46.23; Incongruent: *M* = 888 ms, *SE* = 53) were 3.557 times more likely under the alternative hypothesis (i.e., existence of a priming effect by the local elements). See Figure 3 and Table 2.

Responses to the prime shape discrimination task during the multiple-task block (*M* = .60, *SE* = 0.02) were transformed into a d’_obj_ sensibility measure (*M* = 0.575, *SE* = 0.151) and sent to a Bayesian one sample t-test. Results provided very strong evidence (BF_10_ = 81.106, error % = 1.522 × 10^–5^) favoring group visibility of the primes being above chance level. Data were 81.10 times more likely under the alternative hypothesis (d’_obj_ > 0).

The Bayesian paired samples t-test performed on mean RT data comparing congruent and incongruent trials for PAS-1 reports, showed anecdotal evidence (BF_10_ = 0.846, error % = 4.291 × 10^–5^) against the existence of a congruency effect produced by the local elements in the priming task during the multiple-task block (PAS1-Congruent: *M* = 814 ms, *SE* = 41.15; PAS1-Incongruent: *M* = 827 ms, *SE* = 42.73). See Table 2, and Figure 3.

Last, the results from the Bayesian generative modelling correction to the Greenwald regression showed strong evidence (BF_10_ = 0.072, C.I –0.021/0.017) against a priming effect by the local elements. Data were 13.81 times more likely under the null model (intercept = 0, no subliminal priming effect) compared to a model drawn from a wide normal distribution (see Figure 6B).

### 3.4 Experiment 4: local priming SOA 53 ms

Twenty-three participants took part in Experiment 4 (18 women, age range = 37 years; M = 32.23; SD = 11.98). Two participants were replaced due to an incorrect use of the PAS scale (i.e., more than 10% “clear perception” reports for catch trials in either the multiple-task or the prime visibility blocks). Participants responses were extremely accurate (single-task; Congruent: *M* = .98, *SD* = 0.02, Incongruent: *M* = .97, *SD* = 0.03; dual-task; Congruent: *M* = .98, *SD* = 0.03, Incongruent: *M* = .98, *SD* = 0.02), so only RT data were analyzed.

#### Single-task block analyses

The Bayesian paired samples t-test performed on the individual mean RTs comparing congruent and incongruent trials showed moderate evidence (BF_10_ = 0.120, error % = 0.001) against the existence of a priming effect by the local elements. The RTs obtained in the single task (Congruent: *M* = 568 ms, *SE* = 20.85 ms; Incongruent: *M* = 565 ms, *SE* = 20.02) were 8.33 times more likely under the null hypothesis (i.e., no differences between RTs in congruent and incongruent trials). See Figure 3 and Table 2.

The Bayesian one sample t-test performed on the transformed responses (d’_obj_, *M* = 2.105, *SE* = 0.318) to the prime shape discrimination task during the visibility block (*M* = .79, *SE* = 0.034) provided very strong evidence (BF_10_ = 30866.403 error % = 5.742 × 10^–7^) favoring group visibility being above chance level. Data were 30866 times more likely under the alternative hypothesis (d’_obj_ > 0) than under the null hypothesis (d’_obj_ = 0).

**Figure 7:**
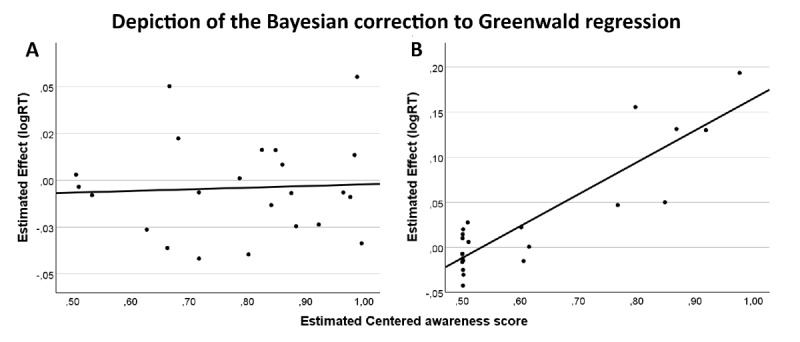
Depiction of the Bayesian correction to Greenwald regression in Experiment 4 (Local SOA 53) in the single-task **(A)** and multiple-task **(B)** blocks. Each circle represents the participant estimated true score. The x-axis represents the centered awareness score, the y-axis represents the estimated effect (incongruent – congruent conditions). The intercept is the expected performance for completely unaware participants.

Last, the results from the Bayesian generative modelling correction to the Greenwald regression showed strong evidence (BF_10_ = 0.076, C.I = –0.028/0.018) against a priming effect by the global shape, indicating that the data were 14.29 times more likely under the null model (no subliminal priming effect) compared to a model drawn from a wide normal distribution (see Figure 7A).

#### Multiple-task block analyses

The distribution of PAS reports during the multiple-task priming block was as follows: PAS1 (no perception of the prime) was reported on 51.4% of the trials, PAS2 (weak perception) on 19% of the trials, PAS3 (almost clear perception) on 12.6% of the trials, and PAS4 (clear perception) on 17% of the trials (see Table 3).

The Bayesian paired samples t-test performed on the individual mean RTs comparing congruent and incongruent conditions in all trials, indicated anecdotal evidence (BF_10_ = 2.882, error % = 9.054 × 10^–5^) favoring the existence of a priming effect by the local elements. The RTs obtained (Congruent: *M* = 951 ms, *SE* = 53.03; Incongruent: *M* = 982 ms, *SE* = 60.02) were 2.88 times more likely under the alternative hypothesis (i.e., existence of a priming effect by the local elements). See Figure 3 and Table 2.

The Bayesian one sample t-test performed on the transformed responses (d’_obj_, *M* = 0.748, *SE* = 0.241) to the prime shape discrimination task during the multiple-task block (*M* = .62, *SE* = 0.03) provided strong evidence (BF_10_ = 16.938, error % = 8.330 × 10^–4^) favoring group visibility of the primes being above chance level. Data were 16.94 times more likely under the alternative hypothesis (d’_obj_ > 0), compared to the null hypothesis (d’_obj_ = 0).

The results from the Bayesian paired samples t-test comparing PAS-1congruent and incongruent trials showed anecdotal evidence (BF_10_ = 0.533, error % = 5.756 × 10^–6^) against the existence of a priming effect produced by the local elements in the priming task during the multiple-task block (PAS1-Congruent: *M* = 957 ms, *SE* = 64.10; PAS1-Incongruent: *M* = 889 ms, *SE* = 53.15). See Table 2, and Figure 3. However, the variability of the PAS reports in the present SOA condition (local priming SOA 53 ms) resulted in five participants having less than ten PAS-1 reports (two participants had no PAS-1 incongruent trials), what in turn led to highly unreliable RTs. Therefore, the results for PAS-1 RTs should be interpreted with caution.

Last, The Bayesian generative modelling correction to the Greenwald regression showed moderate evidence (BF_10_ = 0.110, C.I –0.029/0.0772) against a non-conscious priming effect by the local elements. Data were 9.07 times more likely under the null model (intercept = 0, no subliminal priming effect) compared to a model drawn from a wide normal distribution (see Figure 7B).

### 3.5. General Recognition Theory based analyses: Sensitivity vs Awareness (SvA)

As described in the Data Analysis section, data from the prime shape discrimination task and subjective prime visibility judgments during the multiple-task and visibility blocks were subjected to a GRT model-based analysis of the association between awareness (PAS scale) and the perceptual processing of the prime (prime shape discrimination task). The obtained model was employed to build a SvA curve to characterize the relationship between the perceptual processing of the primes (d’_obj_ for the shape discrimination) and the level of awareness (see section 2.4 for the full description of this analysis).

#### Experiment 1: global priming SOA 40 ms

**Figure 8:**
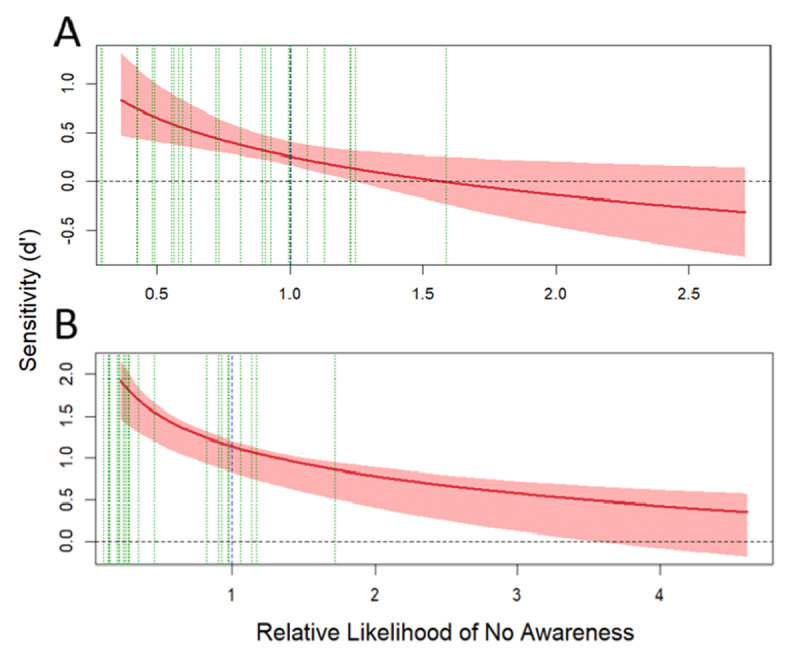
Sensitivity vs. awareness (SvA) curves obtained during the Experiment 1 (Global SAO 40 during the multiple-task **(A)** and visibility blocks **(B)**. The solid red line represents the SvA curve obtained from the best adjusted model, and the lighter red bands represents 95% confidence intervals. The dotted blue line separates regions of relative high likelihood of awareness to the left, and relative low regions o awareness to the right. The dotted green lines are the estimated bounds for each participant. The horizontal black dotted line represents zero sensitivity (d’ = 0) in the prime shape discrimination task.

In the multiple-task block of Experiment 1, after fitting all the sixteen models (the full model along with those derived from the constraints imposed to the full model and their combinations), the model assuming equal variances and perceptual and decisional separability for awareness was found to be the one that represented the data the best (see **Table S1** and **Figure S1** in the supplementary materials), accounting for the 97.85% of the observed response proportions. The main results are depicted in Figure 8A. The x-axis represents the RLNA, and the y-axis represents d’_obj_ computed from the prime shape discrimination task during the multiple-task block. The blue dotted vertical line represents the objective criterion of an ideal observer which divides the x-axis into a region of high likelihood of awareness (left), and a region of low likelihood of awareness to the right. The green dotted vertical lines depict the actual criterion set by each participant. The pattern of the SvA curve (see Pournaghdali et al., 2023 for a complete description of the relationship between different patterns of SvA curves and the awareness/perceptual processing interaction) indicates that perceptual processing of shape is dependent of awareness, as sensitivity drops when RLNA increases. However, there is evidence of nonconscious processing of the global shape: the SvA curve is above a d’_obj_ of zero when it crosses the optimal criterion (blue dotted line) into the region of low likelihood of awareness.

In the visibility block the model assuming equal variances and perceptual and decisional separability for awareness was found to be the one that represented the data the best (see **Table S2** and **Figure S2** in the supplementary materials), accounting for the 95.71% of the observed response proportions. As in the multiple-task analysis, the pattern of the SvA curve indicates (1) that perceptual processing of shape is dependent of awareness, and (2) that there is evidence of nonconscious processing of the global shape: the SvA curve is above a d’_obj_ of zero when RLNA is high (see Figure 8B).

#### Experiment 2: global priming SOA 53 ms

**Figure 9:**
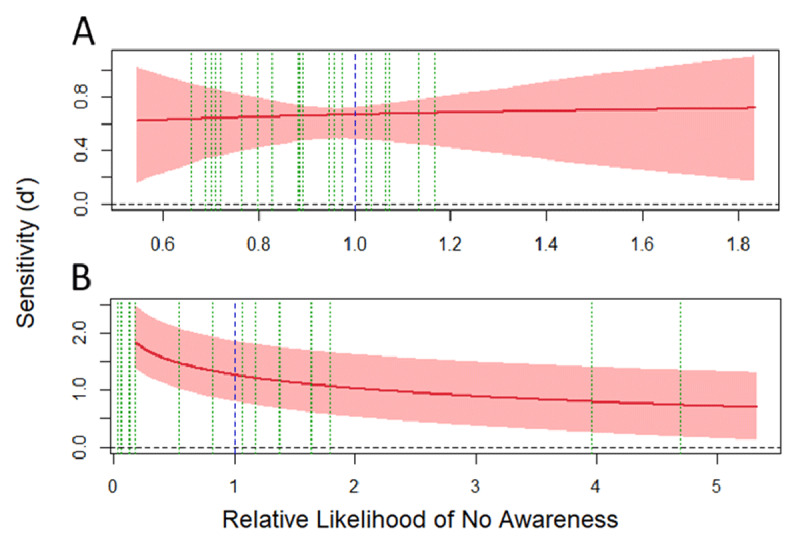
Sensitivity vs. awareness (SvA) curves obtained during the Experiment 2 (Global SOA 53) during the multiple-task **(A)** and visibility blocks **(B)**. The solid red line represents the SvA curve obtained from the best adjusted model, and the lighter red bands represents 95% confidence intervals. The dotted blue line separates regions of relative high likelihood of awareness to the left, and relative low regions o awareness to the right. The dotted green lines are the estimated bounds for each participant. The horizontal black dotted line represents zero sensitivity (d’ = 0) in the prime shape discrimination task.

In the multiple-task block of Experiment 2, the model assuming perceptual and decisional separability for awareness was found to be the one that represented the data the best (see **Table S3** and **Figure S3** in the supplementary materials), accounting for the 95.5% of the observed response proportions. The main results are depicted in Figure 7A. The pattern of the SvA curve indicates: (1) that perceptual processing of shape is independent of awareness, and (2) the existence of nonconscious processing of the global shape, as the SvA curve is above d’_obj_ = 0 when it crosses the optimal criterion (blue dotted line) into the high RLNA region (see Figure 9A).

Regarding the visibility block, the model assuming equal variances and decisional separability for awareness was found to be the one that represented the data the best (see **Table S4** and **Figure S4** in the supplementary materials), accounting for the 97% of the observed response proportions. The pattern of the SvA curve indicates (1) that perceptual processing of shape is dependent of awareness, and (2) that there is evidence of nonconscious processing of the global shape: the SvA curve is above a d’_obj_ of zero when RLNA is high (see Figure 9B).

#### Experiment 3: local priming SOA 40 ms

**Figure 10:**
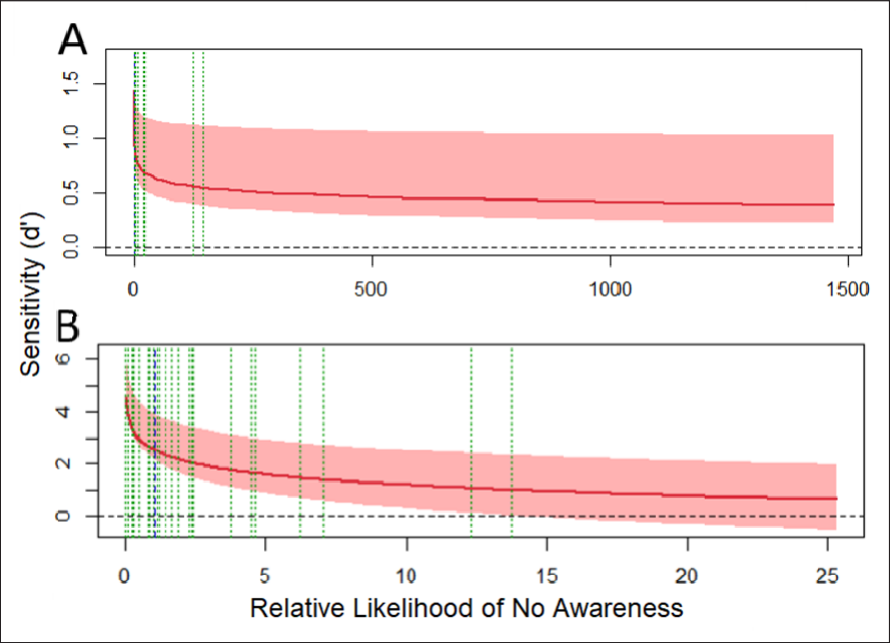
Sensitivity vs. awareness (SvA) curves obtained during the Experiment 3 (Local SOA 40) during the multiple-task **(A)** and visibility blocks **(B)**. The solid red line represents the SvA curve obtained from the best adjusted model, and the lighter red bands represents 95% confidence intervals. The dotted blue line separates regions of relative high likelihood of awareness to the left, and relative low regions o awareness to the right. The dotted green lines are the estimated bounds for each participant. The horizontal black dotted line represents zero sensitivity (d’ = 0) in the prime shape discrimination task.

The analysis of the multiple-task block of Experiment 3 showed that the model assuming perceptual and decisional separability for awareness was found to be the one that represented the data the best (see **Figure S5** and **Table S5** in the supplementary materials), accounting for the 96.14% of the observed response proportions. The main results are depicted in Figure 10-left. The pattern of the SvA curve indicates that the perceptual processing of local elements is dependent of awareness, as sensitivity drops when RLNA increases. However, there is also evidence of nonconscious processing of the local elements: the SvA curve is above a d’_obj_ of zero when it crosses the optimal criterion (blue dotted line) into the region of low likelihood of awareness (see Figure 10A).

On the other hand, in the visibility-block the model assuming decisional separability for awareness was found to be the one that represented the data the best (see **Figure S6** and **Table S6** in the supplementary materials), accounting for the 99.06% of the observed response proportions. The pattern of the SvA curve indicates (1) that perceptual processing of shape is dependent of awareness, and (2) that there is evidence of nonconscious processing of the local elements: the SvA curve is above a d’_obj_ of zero when RLNA is high (see Figure 10B).

#### Experiment 4: local priming SOA 53 ms

**Figure 11:**
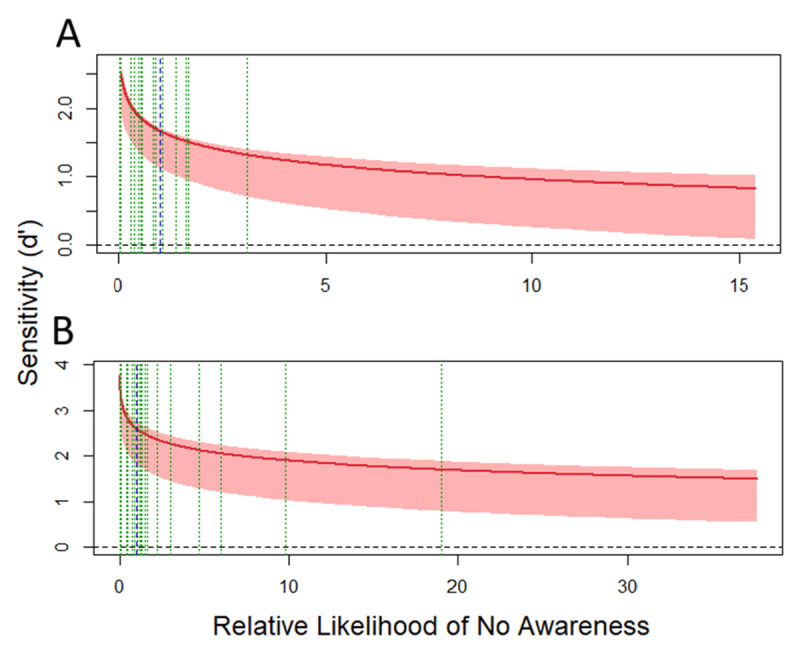
Sensitivity vs. awareness (SvA) curves obtained during the Experiment 4 (Local SOA 53) during the multiple-task **(A)** and visibility blocks **(B)**. The solid red line represents the SvA curve obtained from the best adjusted model, and the lighter red bands represents 95% confidence intervals. The dotted blue line separates regions of relative high likelihood of awareness to the left, and relative low regions o awareness to the right. The dotted green lines are the estimated bounds for each participant. The horizontal black dotted line represents zero sensitivity (d’ = 0) in the prime shape discrimination task.

In the multiple-task block, the model assuming equal variances along with perceptual and decisional separability for awareness was found to be the one that represented the data the best (see **Figure S7** and **Table S7** in the supplementary materials), accounting for the 98.31% of the observed response proportions. The main results are depicted in Figure 11-left. The pattern of the SvA curve indicates that the perceptual processing of local elements is dependent of awareness, as sensitivity drops when RLNA increases. However, there is clear evidence of nonconscious processing of the local elements: the SvA curve is above a d’_obj_ of zero when it crosses the optimal criterion (blue dotted line) into the region of low likelihood of awareness (see Figure 11A).

Last, the analysis of the visibility-block of Experiment 4 showed that the model assuming equal variances and decisional separability for awareness was found to be the one that represented the data the best (see **Figure S8** and **Table S8** in the supplementary materials), accounting for the 98.49% of the observed response proportions. The pattern of the SvA curve indicates (1) that perceptual processing of shape is dependent of awareness, and (2) that there is evidence of nonconscious processing of the local elements: the SvA curve is above a d’_obj_ of zero when RLNA is high (see Figure 11B).

### 3.6. Prime visibility task: d’_obj_ vs. d’_subj_ comparison

To compare prime awareness estimations derived from objective (prime shape discrimination) and subjective (PAS) tasks along the four experiments, objective and subjective awareness criteria were transformed into a common sensitivity measure (d’). Individual mean d’s during the dual task (d’_obj(multiple-task)_ and d’_subj(multiple-task)_) and visibility blocks (d’_obj(visibility)_ and d’_subj(visibility)_) in each experiment were sent to a 2 × 2 Bayesian repeated measures ANOVA with Block (single-task, multiple-task) and d’-type (objective, subjective) as within-subject factors, to assess (1) whether prime awareness estimations differed between objective and subjective measures during the same block, and (2) whether prime awareness estimations differed between the multiple-task block and the visibility block.

Model comparison in both the Global SOA 40 and the Global SOA 53 experiments indicated that the model including Block as main factor explained the data 2.36 (Global SOA 40: Block, BF_10_ = 10.693, error % = 1.603; Block + d’-type: BF_10_ = 4.532, error % = 3.18) and 3.13 (Global SOA 53: Block, BF_10_ = 3.047, error % = 9.814; Block + d’-type: BF_10_ = 0.970, error % = 4.181) times better than a model with Block and d’-type as main factors (both against the null model without factors). Averaging the conclusions from each candidate model weighted by that model’s posterior plausibility (i.e., Bayesian model averaging) showed moderate evidence in favor of Block differences in d’ in the Global SOA 40 experiment (Block, BF_incl_ = 7.648), and only anecdotal evidence favoring Block differences in d’ in the Global SOA 53 experiment (Block, BF_incl_ = 2.093) congruent with the observed overall increase in sensitivity in the visibility block compared to the multiple-task block.

Model comparison in the Local SOA 40 experiment showed that a model including Block and d’-type as main factors and the interaction between Block and d’-type explained the data 5.78 times better than a model with Block and d’-type as main factors (Block, d’-type and Block × d’-type: BF_10_ = 3.661 × 10^7^, error % = 3.279; Block, d’-type: BF_10_ = 6.335 × 10^6^, error % = 2.798). Bayesian model averaging showed strong evidence in favor of block differences on d’ (Block: BF_incl_ = 1.231 × 10^7^), congruent with the increased sensitivity in the visibility block already mentioned. The analysis also showed strong evidence favoring a Block × d’-type interaction (BF_incl_ = 14.020), indicating that differences between objective and subjective d’ measures appeared only in the multiple-task block.

Last, in the Local SOA 53 experiment, the model including Block and d’-type as main factors explained the data 3.62 times better than a model with Block and d’-type as main factors and the interaction between Block and d’-type (Block, d’-type: BF_10_ = 3.132 × 10^7^, error % = 4.284; Block, d’-type and Block × d’-type: BF_10_ = 8.649 × 10^6^, error % = 2.923;). Bayesian model averaging showed strong evidence in favor of block differences on d’ (Block: BF_incl_ = 2.448 × 10^6^), and strong evidence favoring a d’-type effect (BF_incl_ = 10.547), indicating that subjective d’s were higher compared to objective d’s.

**Figure 12:**
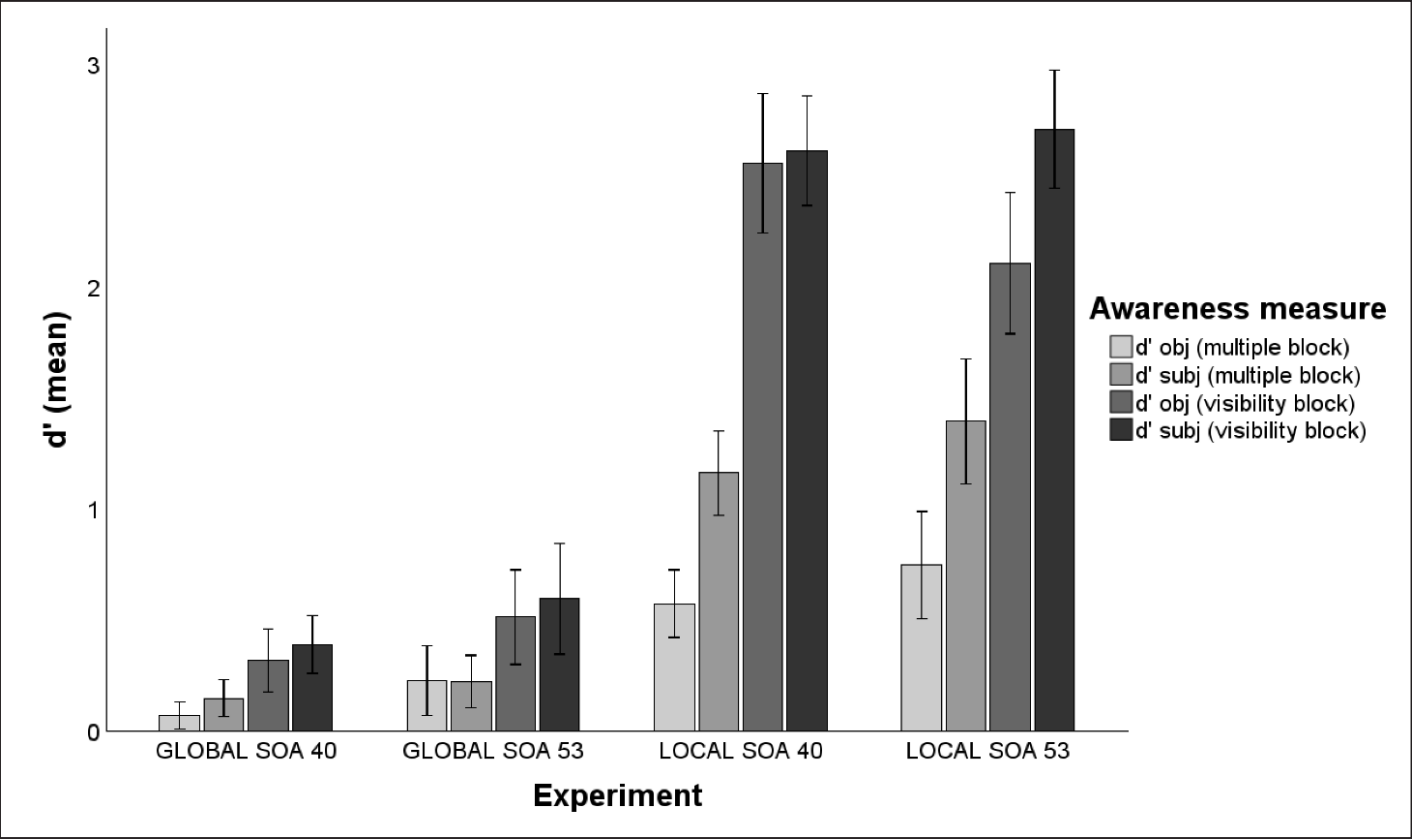
Sensitivity measures (d’) obtained during the multiple-task and visibility blocks (d’_obj_ and d’_subj_).

Overall, these results seem to be driven by an overall increased visibility of the primes during the visibility block, compared to the multiple-task priming block, along with a tendency for subjective measures to be higher than objective measures, more pronounced in the local priming experiments. (see Table 3 and Figure 12).

Finally, to analyze the degree to which d’_obj_ and d’_sub_ were related within the same task and whether a given sensitivity measure was consistent across the different blocks, we conducted Bayesian linear correlations between awareness measures within the multiple-task (d’_obj(multiple-task)_ and d’_subj(multiple-task)_) and visibility blocks (d’_obj(visibility)_ and d’_subj(visibility)_), and between the same measure across different blocks (d’_obj(multiple-task)_ and d’_obj(visibility)_; d’_subj(multiple-task)_ and d’_subj(visibility)_). The results showed overall strong correlations within measures/between blocks and between measures/within blocks, indicating great consistency across the different measures of awareness collected (see Table 4 for a summary; see also the supplementary materials for a complete correlation matrix between awareness measures).

**Table 4:** d’ values for objective and subjective sensitivity measures of prime shape discrimination. Bayes factor (BF_10_) and Pearson’s correlation coefficients (r) for the relevant comparisons between sensitivity measures across all four experiments. * BF_10_ > 10, ** BF_10_ > 30, *** BF_10_ > 100.


EXPERIMENT	d’ VALUES	CORRELATION	BF_10_	PEARSON’S *r*

**Global SOA40 ms**	d’_obj(multiple-task)_ = 0.073	d’_obj(multiple-task)_ – d’_subj(multiple-task)_	5.259	0.463

	d’_obj(visibility)_ = 0.319	d’_obj(visibility)_ – d’_subj(visibility)_	289.221	0.694***

	d’_subj(multiple-task)_ = 0.151	d’_obj(multiple-task)_ – d’_obj(visibility)_	1865.976	0.757***

	d’_subj(visibility)_ = 0.392	d’_subj(multiple-task)_ – d’_subj(visibility)_	824.921	0.731***

**Global SOA53 ms**	d’_obj(multiple-task)_ = 0.230	d’_obj(multiple-task)_ – d’_subj(multiple-task)_	15.906	0.625*

	d’_obj(visibility)_ = 0.515	d’_obj(visibility)_ – d’_subj(visibility)_	1916.471	0.814***

	d’_subj(multiple-task)_ = 0.226	d’_obj(multiple-task)__–_ d’_obj(visibility)_	163.048	0.737***

	d’_subj(visibility)_ = 0.596	d’_subj(multiple-task)_ – d’_subj(visibility)_	25258.694	0.869***

**Local SOA40 ms**	d’_obj(multiple-task)_ = 0.575	d’_obj(multiple-task)_ – d’_subj(multiple-task)_	56.915	0.622**

	d’_obj(visibility)_ = 2.551	d’_obj(visibility)_ – d’_subj(visibility)_	8642.373	0.781***

	d’_subj(multiple-task)_ = 1.162	d’_obj(multiple-task)_ – d’_obj(visibility)_	203.543	0.674***

	d’_subj(visibility)_ = 2.609	d’_subj(multiple-task)_ – d’_subj(visibility)_	227.081	0.678***

**Local SOA53 ms**	d’_obj(multiple-task)_ = 0.748	d’_obj(multiple-task)_ – d’_subj(multiple-task)_	293.053	0.720***

	d’_obj(visibility)_ = 2.105	d’_obj(visibility)_ – d’_subj(visibility)_	47.654	0.646**

	d’_subj(multiple-task)_ = 1.395	d’_obj(multiple-task)_ – d’_obj(visibility)_	2288.726	0.782***

	d’_subj(visibility)_ = 2.706	d’_subj(multiple-task)_ – d’_subj(visibility)_	9865.952	0.816***


## 4. Discussion

The present study employed a masked priming design with hierarchical visual stimuli similar to the ones employed by Sabary et al. (2020), and Jimenez et al. (2023) in two different masking conditions (SOA 40 and 53 ms), to explore the relationships between perceptual organization and consciousness, and particularly whether global and local shapes can be unconsciously processed. To this end, we compared three alternative approaches: the classical dissociation paradigm, and the two newly proposed Bayesian generative and General Recognition Theory models. Crucially, we implemented an exhaustive, integrative procedure in which objective and subjective, as well as offline and online measures of awareness were combined in a within-subjects design, allowing us a thorough comparison of prime sensitivity (d’) for the different combinations of awareness measures.

Overall, the pattern of results obtained can be summarized around three key points: (1) The classic analysis strategies associated to the masked priming paradigm showed mixed evidence regarding the unconscious processing of the primes, that seemed to be influenced by the type of priming (local vs global) and the way in which awareness measures were collected (online vs offline). Interestingly, when a Bayesian generative model (Goldstein et al., 2022) was fitted to the data, any evidence in favor of prime related facilitation effects seemed to vanish across all conditions and experiments. (2) Conversely, when the unconscious processing of the prime was assessed directly through a general recognition theory (GRT) model-based analysis that measured the association between awareness and perceptual processing, our results indicated that primes were processed even when the relative likelihood of no awareness was high. (3) Finally, when converted to a common sensitivity measure (d’), objective and subjective measures of awareness collected during the multiple-task (online) and visibility blocks (offline), showed similar values within the same task, a high degree of correlation both within and between tasks and, importantly, both were sensitive to the differences in prime visibility associated to the attentional variations due to the different task demands (an overview of the main results can be found in Table 5). Each of these points will be discussed in more detail below.

**Table 5:** Summary of the results according to the different types of analysis performed.


ANALYSIS METHOD	MAIN RESULTS

**Classical dissociation paradigm:** *(Objective awareness measures)*	Evidence favoring priming effects for the global shape in Experiments 1 (strong) and 2 (anecdotal) during the single-task block. Evidence against priming effects for the local elements in Experiments 3 and 4 (moderate) during the single-task block. No evidence for priming effects for the global shape in Experiments 1 and 2 during the multiple task block Evidence favoring priming effects for the local elements in Experiments 3 and 4 (moderate) during the multiple-task block. Increased RT and variability during the multiple-task block compared to the single-task block All d’ > 0 except in the multiple-task block of Experiment 1

**Perceptual awareness scale (PAS-1):** *(Subjective awareness measures)*	Evidence against the existence of unconscious priming effects for the global shape in the multiple-task block of Experiment 1 (moderate) and 2 (anecdotal) Evidence against the existence of unconscious priming effects for the local elements in the multiple-task block of Experiment 3 (anecdotal) and 4 (anecdotal). No evidence of priming effects when only PAS-1 (non-conscious) trials were analyzed

**Bayesian regression** *(Objective awareness measures)*	Evidence against the existence of unconscious priming effects for the global shape in the single-task block of Experiment 1 (moderate) and 2 (moderate). Evidence against the existence of unconscious priming effects for the local elements in the single-task block of Experiment 3 (strong) and 4 (strong). Evidence against the existence of unconscious priming effects for the global shape in the multiple-task block of Experiment 1 (moderate) and 2 (moderate). Evidence against the existence of unconscious priming effects for the local elements in the multiple-task block of Experiment 3 (strong) and 4 (moderate).

**GRT-based SvA curves** *(Subjective awareness measures)*	Greater than 0 sensitivity (d’) when RLNA was high (>1) for the global shapes in Experiment 1 (≈ 0.25) and 2 (≈ 0.6) during the multiple-task block Greater than 0 sensitivity (d’) when RLNA was high (>1) for local elements in Experiment 3 (≈ 0.75) and 4 (≈ 1.7) during the multiple-task block Greater than 0 sensitivity (d’) when RLNA was high (>1) for the global shapes in Experiment 1 (≈ 1.25) and 2 (≈ 1.5) during the visibility block Greater than 0 sensitivity (d’) when RLNA was high (>1) for local elements in Experiment 3 (≈ 2.5) and 4 (≈ 2.7) during the visibility block

**Objective VS subjective awareness measures comparison** *(d’_obj_ vs d’_subj_)*	Greater d’ values (both objective and subjective during the visibility block (full attention to the primes), compared to the multiple-task block (divided attention between primes and probes) in all experiments. Overall greater d’ values when assessed by means of subjective awareness measures, compared to objective awareness measures. Overall strong correlations between objective (d’_obj_) and subjective (d’_subj_) measures of awareness within the same block (multiple-task and visibility blocks respectively) Overall strong correlations within objective (d’_obj_) measures of awareness collected in different blocks (multiple-task and visibility blocks respectively) Overall strong correlations within subjective (d’_subj_) measures of awareness collected in different blocks (multiple-task and visibility blocks respectively)


Indeed, when the data were analyzed following the classical masked priming (dissociation) paradigm (Reingold & Merikle, 1988), we found strong evidence of priming effects for the global shape in Experiment 1 (SOA-40) during the single-task block, but only anecdotal evidence of priming effects in Experiment 2 (SOA-53), also during the single-task block. No evidence of priming effects by the global shape were found in the multiple-task block in the first two experiments. Interestingly, the pattern of results reversed for the local priming conditions. Moderate evidence against the existence of a priming effect was found in Experiments 3 (SOA-40) and 4 (SOA-53) in the single-task block, whereas in the multiple-task block, moderate evidence of the existence of a priming effect was found for both SOA conditions (see Figure 3 and Table 2). This result has been surprising, since, in line with previous works (Jimenez et al., 2023; Kiefer et al., 2023), we expected the inclusion of a multiple-task condition (the probe discrimination task plus the online awareness measures collected in each trial) to interfere with the operations underlying masked priming, presumably due to the split of attentional resources between the prime and probe (Ansorge et al., 2011; Avneon & Lamy, 2018; Jimenez et al., 2023; Kiefer et al., 2023). In fact, this is what seemed to be happening, as evidenced by the substantial increase in the RTs and their variability during the multiple-task block in all experiments. One tentative explanation for these conflicting results could arise from the interaction between the greater visibility of the primes during the local priming conditions (as shown by the higher objective and subjective awareness measures, see Figure 12 and Table 3) and differences in attentional resources devoted to the prime during the single and the multiple-task blocks. In the global priming conditions (Experiments 1 and 2) the level of sensitivity (d’_obj_ and d’_subj_ < 0,5) to the masked primes during the multiple-task block was very low, even though the amount of attentional resources devoted to the masked prime task was higher compared to the single block (where participants simply ignored the prime as it was irrelevant to the task). In this situation of extremely low prime visibility, the inclusion of the multiple-task condition would have resulted in a reduction of the resources allocated to the probe discrimination task, eliminating the congruency effects found during the single task. Conversely, in the local priming conditions (Experiments 3 and 4) the sensitivity to the prime during the multiple-task block was remarkably higher compared to the global priming conditions (see Figure 12 and Table 3). In this scenario, the higher visibility of the primes could be responsible for the support found towards a priming effect in the multiple-task block, as it would exert a stronger effect than the possible attenuation resulting from the reduced attentional resources to the probe due to the dual task conditions. Indeed, this is congruent with the vanishing of the priming effects in the local condition during the multiple-task block when only PAS-1 trials were analyzed (see Figure 3 and Table 2), indicating that the priming effects were driven mostly by conscious trials. Our results are also congruent with previous studies on the unconscious processing of hierarchical stimuli. For example, Sabary et al. (2020) found priming effects by the local elements of hierarchical stimuli in a priming task in which online subjective awareness measures were collected in each trial, but did not found priming effects by the global shape in the same condition. On the contrary, Jimenez et al. (2023) found clear priming effects by the global shape in a single-task condition that disappeared when subjective awareness measures where collected in a trial-by-trial basis (dual-task condition). These results also emphasize the need for individual difference studies, as there appear to be large differences in both the unconscious effects found, and the visibility of the masked stimuli among participants, as can be seen from the individual data in the regression analyses (Figures 4, 6, 8 and 10, black dots) and the SvA curves (Figures 5, 7, 9 and 11, dotted green lines).

As we discussed in the introduction section, classical approaches to the analysis of the unconscious effects of masked priming designs have faced several challenges. Particularly, when using objective performance measures of awareness within the classical dissociation paradigm, one might wonder whether prime related effects might be a consequence of the average level of awareness being above zero or, even if the level of performance in the awareness measures does not exceeds the chance level, the effects found are due to some participants whose awareness levels are particularly high (Rouder et al., 2007; Sandberg et al., 2010; Shanks, 2017; Timmermans & Cleeremans, 2015). An analogous situation occurs when online subjective measures are collected in each trial. In this case, priming effects could be derived from trials in which participants were aware of the prime (e.g., PAS 2, 3, and 4 reports). In agreement with these concerns, we found that sensitivity levels were above chance level in all experiments and conditions except for d’_obj_ during the multiple-task block in the Global SOA-40 experiment (d’_obj_ ranging from 0.07 to 2.11; d’_subj_ ranging from 0.15 to 2.71, see Figure 12 and Table 3). Therefore, to rule out that priming effects found were due to a limited number of “conscious” participants or “conscious” trials, and at the same time avoiding post-hoc data selection and potential regression to the mean effects (Rothkirch et al., 2022; Shanks, 2017; Yaron et al., 2023), we implemented a Bayesian generative regression model recently developed by Goldstein et al., (2022). This method estimates intercept in a regression model that represents the priming effect for an ideal observer whose awareness (estimated by the performance achieved in the objective prime shape discrimination task) is equal to zero. Interestingly, when using this method, we found moderate to strong evidence favoring the null hypothesis and against the unconscious processing of the primes in all priming, task and SOA conditions (see Figures 4, 6, 8, and 10; and Figures S9 to S16 in the supplementary materials). These results are in line with an explanation of the priming effects based on a subset of participants that are at least partially aware of the masked prime, while those who are not have negligible priming effects, an account that has been defended by some authors in light of the volatility that unconscious priming effects usually present throughout the scientific literature (Newell & Shanks, 2014; Shanks et al., 2021; Vadillo et al., 2022). However, it is important to point out that while the regression model estimated for the multiple-task block was based on an objective-online measure (and, therefore, collected under the same conditions in which the priming task was performed), the estimated model for the single-task block was collected during the separate visibility block (objective-offline). Hence, we cannot rule out that the absence of an unconscious effect showed by the regression model in the single-task block derives from an overestimation of the level of awareness of the prime during the visibility block (remember that, in the single-task block, the participants just ignore the prime and focused on the probe, while in the visibility block participants’ attentional resources were fully centered on the prime). This conclusion is supported by the lower awareness estimations during the multiple-task block in which attentional resources were split between the prime and the probe. Interestingly, this brings us to a paradoxical situation within the dissociation paradigm. Either we measure awareness concurrently with the task, meeting the criterion of immediacy, but compromising the reliability of the indirect measure of performance and the awareness assessment (Newell & Shanks, 2014). Or, alternatively, we measure awareness separately, improving reliability but renouncing to the immediacy criterion and, to some extent, to part of the validity of our measures (Bengson & Hutchison, 2007; Lähteenmäki et al., 2015; Shanks & John, 1994). This would resemble an *uncertainty principle* (Heisenberg, 1927) for the study of (un)consciousness and exposes the researchers to an unavoidable dilemma that we will have to deal with in future research on this topic.

All the results discussed above rely on the indirect examination of the unconscious perception of the local elements or the global shape of the stimuli by means of the comparison between congruent and incongruent prime-probe responses. However, the paradigm implemented in this study also makes it possible to assess the unconscious processing of the masked stimulus directly, as participants performed simultaneously an objective prime shape discrimination task and a subjective visibility judgement using the PAS scale during the multiple-task and the visibility blocks. This direct evaluation has been employed in previous studies (Heeks & Azzopardi, 2015; Lähteenmäki et al., 2015; Pournaghdali et al., 2023; Szczepanowski & Pessoa, 2007) and, analogously to the classical masked priming paradigm, it also involves a dissociation between two different measures. However, this dissociation now involves a comparison between a subjective awareness report and the performance in an objective forced-choice task (either detection or discrimination). This change is not trivial, as it implies a shift in the theoretical assumptions about what constitutes conscious and unconscious processing. Particularly, we move from using d’ in the forced-choice discrimination task as a measure of awareness (implying that unconscious processing is only possible if d’ = 0), to using it as a measure of perceptual processing (implying that a d’ > 0 followed by a subjective report of no awareness is necessary to assume unconscious processing of the stimuli). In other words, we switch from a more conservative objective threshold to a subjective threshold for which unconscious processing constitutes a state that falls between the two (Cheesman & Merikle, 1984; Heeks & Azzopardi, 2015; Jimenez et al., 2024; Lamme, 2020; Michel, 2023). To analyze the existence of unconscious processing under these assumptions, we employed the method developed by Pournaghdali et al. (2023) based on the GRT framework (see Ashby & Soto, 2015) that we described in the introduction section. According to the results depicted in Figure 5, Figure 7, Figure 9 and 11, the capacity to discriminate between different shapes at the local and the global level persists in the absence of awareness, although the strength of the discrimination depends on its level (i.e., as the RLNA of the stimulus increases, the performance in the prime shape discrimination task decreases). This contrast with the results obtained using indirect methods to estimate unconscious processing which, after removing possible conscious effects (either via a regression model or by selecting unconscious trials), pointed to the absence of unconscious processing of the primes. Nonetheless, the decoupling between the results obtained through different methodologies is in accordance with the previously described distinction between objective and subjective awareness thresholds (Merikle et al., 2001). It is often assumed that the objective threshold is more restrictive than the subjective threshold, and consequently that it is associated with a more conservative estimate of participant’s awareness. Therefore, the fundamental question is to determine what exactly each of these thresholds is measuring, a problem that has been heavily debated and is still a matter of discussion (Jimenez et al., 2024; Michel, 2023; Timmermans & Cleeremans, 2015). One possible solution is to consider the different thresholds as different stages in the visual processing stream: a *fully unconscious state*, which refers to stimuli below the objective threshold of visibility (e.g., d’ = 0); a *subjectively unconscious state* denoting subjectively unseen stimuli (e.g., d’ > 0 together with a PAS-1 report); and a *subjectively conscious state* reserved to those stimuli that surpass both thresholds (Lamme, 2020). This classification somewhat resembles the distinction between phenomenal and access consciousness proposed by Block (2007). In Block’s proposal, it could be possible to think about phenomenal consciousness (a conscious state that cannot be accessed or reported by the subject) as a different interpretation of the subjectively unconscious state proposed by Lamme (2020). Once again, the question brings us back to the very definition of awareness and the underlying phenomena that we are trying to measure, or whether it is possible to have sensitivity at the subject level in the absence of a *feels like something* experience. A final critical point to note is that the SvA analysis also showed differences in the strength of the unconscious processing as a function of the type of task performed. As shown in Figure 5, Figure 7, Figure 9, and 11, the discrimination capacity (d’) when the RLNA criterion is crossed (blue dotted line) is higher during the visibility block (right side of the figures) compared to the multiple-task block (left side). This is consistent with our expectations about the effect of the level of attention devoted to the primes, and with the differences found in the common sensitivity measures (d’) calculated to compare awareness measures and which will be discussed in the following paragraphs.

Our last purpose was to compare the distinct types of awareness measures collected according to the two dimensions proposed at the Introduction: the objective-subjective and the online-offline axes. To achieve this goal we converted each awareness measure into a common sensitivity metric (d’) derived from the signal detection theory (Kunimoto et al., 2001; Maniscalco & Lau, 2012, 2014; Szczepanowski & Pessoa, 2007). The results suggest two different but complementary conclusions. First, online measures tend to give lower estimates of awareness than offline measures, and the same occurs when comparing objective measures against subjective measures (see Figure 12 and Table 3). These results are not surprising as they reflect the differences in the unconscious processing of the primes between tasks, and emphasize the relevance of the attentional resources devoted to the processing of the masked stimuli in the emergence of awareness (De Brigard & Prinz, 2010; Koch & Tsuchiya, 2012) and also in the degree to which unconscious stimuli are processed (Kanai et al., 2006; Maier & Tsuchiya, 2021; Naccache et al., 2002). They are also in line with the objective and subjective threshold account that we discussed above (Lamme, 2020), as indicated by the lower sensitivity values obtained from the objective awareness measures. The second conclusion is derived from the high correlations found between objective and subjective awareness measures within the same block, and between the same awareness measures across different blocks. This high level of correlation indicates that, irrespective of the absolute value of each measure, participants are highly consistent in their awareness estimation across measures and tasks. Although at first glance they appear to conflict with the already discussed differences between measures and tasks, the two results can be easily integrated within a multiple stage processing account (Lamme, 2020). According to this view, the absolute differences found between measures and task demands would correspond with the different demands of processing necessary to reach the objective and subjective awareness threshold, which would also be influenced by the level of attention paid to the stimuli. On the other hand, the high correlation between measures within the same task and across different tasks within the same measure, would be interpreted as evidence in favor of their validity, or at least indicating that the same construct underlies both measures.

In sum, our results indicate that the unconscious processing of the local and/or the global form in hierarchical stimuli heavily depends on (1) the type of measure collected, (2) how and when the measure was collected, and (3) the theoretical assumptions under which a given measure is analyzed and interpreted. However, none of the awareness measures, the methods employed to collect them, or the analysis strategies conducted, are entirely satisfactory, as all imply the renunciation to some of the prerequisites needed for a proper measurement of (un)awareness, or the loss of useful information about the effects associated with the (un)conscious processing of the stimulus. Therefore, it seems clear that the only way to achieve a better understanding of the mechanisms underlying conscious and unconscious processing is to conduct an experimental *tour de force* through different measures and methods, that allow us to integrate the evidence obtained into a comprehensive framework on how to identify, differentiate and quantify the conscious and the unconscious.

## Data Accessibility Statement

The methods used and the data analyzed in the present study are available in the Open Science Framework (OSF) repository through the following link: https://doi.org/10.17605/OSF.IO/G3AKU.

## Additional File

The additional file for this article can be found as follows:

10.5334/joc.411.s1 Supplementary Materials. 1. Results of the GRT model comparison and selection; 2. Estimated GRT-wIND models for each experiment and block; 3. Estimated intercepts and BFs for the Bayesian generative regression models; 4. Comparison between the RTs in the single-task and multiple-task blocks; 5. Correlation matrices between sensitivity measures (d’).

## Footnotes

A 2AFC task refers to a task in which the observer is presented with two alternative stimuli simultaneously (or sequentially in a two-interval forced choice, 2IFC task) and forced to choose one based in a previously instructed criterion. Although the term is sometimes used in a more loosely form to describe a task where the subject views a single stimulus and must perform one of two responses, this use is not correct and has been discouraged by multiple authors (Hautus, 2015; Hautus et al., 2021; Schneider & Parker, 2009). It is important to note that our paradigm does not include the presence of two simultaneous stimuli and, therefore, does not constitute a 2AFC task (see Hautus, 2015 for an analysis of the implications of the use of a 2AFC task on the estimation of d’). Therefore, we will use the terms ‘prime discrimination task’ and ‘probe discrimination task’ to refer to the tasks employed in the present study.

The criterion content problem was first introduced by Kahneman (1968) to highlight that observers base their perceptual (or metacognitive) decisions on a given set of features, which may not necessarily be the ones relevant to the task. This leads to the criterion content fallacy: concluding that observers are conscious of the task-relevant features only because they are conscious of something (Michel, 2023).

The statistical phenomenon of regression to the mean refers to the fact that due to the post hoc selection of a sub-sample of participants (double-dipping), the expected measurement error is no longer random. Instead, selecting the subgroup with the lowest awareness scores increases the probability of underestimation of awareness, as errors are expected to be biased towards the upper end of the awareness level.

In fact, the regression method was not originally intended to overcome the post-hoc selection problem, but to address other problems related to the demonstration of the existence of unconscious processing by indirect methods, specifically the assumptions made for the direct measures, a detailed explanation of which is beyond the scope of this paper (see Greenwald et al.,1995). However, since it allows avoiding post-hoc selection of participants, it has been used by other authors for this purpose (Goldstein et al., 2022; Klauer et al., 1998).

Specifically, we tested a minimum of 20 participants per experiment, corresponding to 2560 observations per condition, which exceeds the minimum observations per condition recommended by Brysbaert and Stevens (2018) for properly powered reaction time experiments.

Bayesian tests were conducted using a default scale parameter of 0.707 (√2/2) for a Cauchy prior centered on zero.

In this method, the proportion of correct responses (p-correct) in the prime shape discrimination task is employed as awareness score. As the expected chance-level performance in a two alternative discrimination task is 0.5, centered awareness scores were computed by subtracting 0,5 to the proportion of correct responses in the task for each participant. Thus, those participants that performed at chance-level have a centered score of 0.

See Goldstein et al. (2022) and Jimenez et al. (2023) for a detailed explanation of the Bayesian method employed in the regression analysis.

Initially, RT data in the multiple-task block were intended to be analysed by means of a 2 × 4 repeated measures Bayesian ANOVA with congruency (congruent vs incongruent) and PAS (1: No perception; 2: Weak perception; 3: Almost clear perception, and 4: Clear perception) as within-subjects factors. However, considering the small datasets for PAS-3 and PAS-4 conditions in the global priming experiments, the limited number of participants that would entered the Bayesian ANOVA after listwise deletion due to missing values, especially PAS-2, PAS-3, and PAS-4, and also that the main objective of the study was to explore the priming effects in the absence of awareness, only the individual mean RT data for PAS-1 reports (no perception of the primes) finally entered this analysis.

To adapt the PAS scale used in our study to the 2 × 2 two-dimensional space of the GRT, we collapsed the 4 possible visibility judgments of the PAS scale into two categories namely: unseen (PAS-1) and seen (PAS-2, 3 and 4). Note that this is a quite conservative awareness criterion, as we consider as unaware trials only those in which participants reported no perception at all. To avoid assuming any conscious trial as an unconscious one, PAS-2 judgments were considered as conscious trials, even though this judgement was reserved for situations where, despite having seen something, none of the relevant characteristics of the stimulus could be identified.

Model estimation started with a full model in which all parameters were fitted to the data. Then we sequentially constrained the model by fixing the following parameters: (1) All variances were fixed, (2) it was assumed that decisions about the identity of the prime (square or diamond) were not influenced by the level of awareness of the primes, (3) it was assumed that decisions about the awareness of the primes were not influenced by their identity (square or diamond), and (4) it was assumed that the distribution of awareness judgments for a given stimulus was not influenced by the identity of that stimulus. These constraints and their combinations were combined to construct 16 different models that were then compared (see Pournaghdali et al., 2023 for a detailed explanation).
