# Supplementary material for: In Search of an Integrative Method to Study Unconscious Processing: An Application of Bayesian and General Recognition Theory Models to the Processing of Hierarchical Patterns in the Absence of Awareness
Source: J Cogn. 2025 Jan 6;8(1):6. doi: 10.5334/joc.411 (PMC11720486; doi:10.5334/joc.411)
Supplement: Supplementary Materials. — 1. Results of the GRT model comparison and selection; 2. Estimated GRT-wIND models for each experiment and block; 3. Estimated intercepts and BFs for the Bayesian generative regression models; 4. Comparison between the RTs in the single-task and multiple-task blocks; 5. Correlation matrices between sensitivity measures (d’). [file joc-8-1-411-s1.pdf]

Supplementary Materials

1. Results of Model Comparison and Selection

The tables S1 to S8 summarize the results of model comparison with AIC. The first column is the number assigned to each model in order to identify it. The second column is the set of assumptions implemented in the model. The third is the log-likelihood of the model at the best-fitting parameter values, and the fourth column is the model's AIC value. The fifth column shows the probability that the model is the best among those included in the comparison (the sixteen models fitted). En each table VAR1 means equal variance, DS(Awr) means decisional separability for awareness, DS(Shp) means decisional separability for shape, DS means decisional separability for both dimensions (shape and awareness), PS(Awr) means perceptual separability for awareness, and "full" represents the unconstrained model without any assumptions.

Model number	Assumptions	Log-likelihood	AIC	AIC weight
2	VAR1, DS(Awr), PS(Awr)	-6632.926	13699.57	.693
8	DS(Awr), PS(Awr)	-6623.955	13701.21	.305
5	DS(Awr)	-6622.598	13711.99	0
1	VAR1, DS(Awr)	-6653.523	13747.20	0
10	VAR1, DS(Shp), PS(Awr)	-6671.766	13777.25	0
9	VAR1, DS(Shp)	-6668.934	13778.03	0
12	DS(Shp), PS(Awr)	-6668.041	13789.38	0
11	DS(Shp)	-6666.651	13800.09	0
15	PS(Awr)	-6598.902	13831.74	0
6	DS, PS(Awr)	-6601.724	13837.38	0
16	full	-6607.017	13866.80	0
7	DS	-6609.450	13871.66	0
14	VAR1, PS(Awr)	-6644.345	13895.47	0
13	VAR1	-6642.192	13900.08	0
4	VAR1, DS, PS(Awr)	-6647.221	13901.22	0
3	VAR1, DS	-6647.478	13910.65	0

TableS1: Summary of the GRT models fitted during the multiple-task block in Experiment 1 (Global SOA-40)

Model number	Assumptions	Log-likelihood	AIC	AIC weight
2	VAR1, DS(Awr), PS(Awr)	-7020.386	14474.49	1
5	DS(Awr)	-7017.108	14501.01	0
1	VAR1, DS(Awr)	-7033.806	14507.77	0
8	DS(Awr), PS(Awr)	-7027.359	14508.02	0
4	VAR1, DS, PS(Awr)	-7002.933	14612.65	0
3	VAR1, DS	-7007.217	14630.13	0
14	VAR1, PS(Awr)	-7015.595	14637.97	0
7	DS	-6993.550	14639.86	0
13	VAR1	-7017.685	14651.06	0
16	full	-7004.253	14661.27	0
15	PS(Awr)	-7029.008	14691.95	0
6	DS, PS(Awr)	-7033.136	14700.21	0
12	DS(Shp), PS(Awr)	-7187.273	14827.84	0
11	DS(Shp)	-7193.019	14852.83	0
9	VAR1, DS(Shp)	-7211.462	14863.08	0
10	VAR1, DS(Shp), PS(Awr)	-7221.772	14877.26	0

TableS2: Summary of the GRT models fitted during the visibility block in Experiment 1 (Global SOA-40)

Model number	Assumptions	Log-likelihood	AIC	AIC weight
8	DS(Awr), PS(Awr)	-5994.548	12391.56	.96
5	DS(Awr)	-5990.837	12397.92	.4
2	VAR1, DS(Awr), PS(Awr)	-6026.830	12436.22	0
1	VAR1, DS(Awr)	-6036.265	12461.62	0
11	DS(Shp)	-6037.052	12490.35	0
3	VAR1, DS	-5975.196	12494.68	0
7	DS	-5965.926	12514.07	0
12	DS(Shp), PS(Awr)	-6056.037	12514.53	0
6	DS, PS(Awr)	-5978.078	12519.07	0
15	PS(Awr)	-5980.847	12524.60	0
16	full	-5971.380	12524.98	0
13	VAR1	-5992.015	12528.32	0
14	VAR1, PS(Awr)	-5997.556	12530.33	0
10	VAR1, DS(Shp), PS(Awr)	-6077.077	12536.71	0
9	VAR1, DS(Shp)	-6085.202	12559.50	0
4	VAR1, DS, PS(Awr)	-6034.579	12604.38	0

TableS3: Summary of the GRT models fitted during the multiple-task block in Experiment 2 (Global SOA-53)

Model number	Assumptions	Log-likelihood	AIC	AIC weight
1	VAR1, DS(Awr)	-6556.953	13503.00	1
2	VAR1, DS(Awr), PS(Awr)	-6573.029	13528.61	0
5	DS(Awr)	-6562.685	13541.62	0
8	DS(Awr), PS(Awr)	-6584.458	13571.38	0
13	VAR1	-6529.646	13603.58	0
3	VAR1, DS	-6534.869	13614.02	0
4	VAR1, DS, PS(Awr)	-6541.102	13617.43	0
14	VAR1, PS(Awr)	-6546.090	13627.40	0
16	full	-6559.681	13701.58	0
15	PS(Awr)	-6576.683	13716.28	0
7	DS	-6571.851	13725.92	0
6	DS, PS(Awr)	-6593.142	13749.19	0
12	DS(Shp), PS(Awr)	-6731.968	13866.40	0
11	DS(Shp)	-6730.599	13877.45	0
10	VAR1, DS(Shp), PS(Awr)	-6853.828	14090.21	0
9	VAR1, DS(Shp)	-6861.615	14112.32	0

TableS4: Summary of the GRT models fitted during the visbility block in Experiment 2 (Global SOA-53)

Model number	Assumptions	Log-likelihood	AIC	AIC weight
8	DS(Awr), PS(Awr)	-7738.123	15980.52	1
2	VAR1, DS(Awr), PS(Awr)	-7763.346	16011.63	0
5	DS(Awr)	-7748.420	16014.39	0
1	VAR1, DS(Awr)	-7773.892	16039.09	0
3	VAR1, DS	-7696.220	16079.68	0
14	VAR1, PS(Awr)	-7704.851	16088.15	0
4	VAR1, DS, PS(Awr)	-7715.019	16108.48	0
13	VAR1	-7713.413	16114.07	0
15	PS(Awr)	-7708.471	16122.15	0
6	DS, PS(Awr)	-7712.402	16130.01	0
7	DS	-7705.589	16134.84	0
16	full	-7709.527	16142.72	0
11	DS(Shp)	-7852.052	16221.66	0
12	DS(Shp), PS(Awr)	-7874.443	16253.17	0
9	VAR1, DS(Shp)	-7884.674	16260.66	0
10	VAR1, DS(Shp), PS(Awr)	-7899.056	16283.05	0

TableS5: Summary of the GRT models fitted during the multiple-task block in Experiment 3 (Local SOA-40)

Model number	Assumptions	Log-likelihood	AIC	AIC weight
5	DS(Awr)	-5300.163	11117.88	.998
8	DS(Awr), PS(Awr)	-5313.540	11131.36	1
1	VAR1, DS(Awr)	-5321.165	11133.64	0
2	VAR1, DS(Awr), PS(Awr)	-5333.318	11151.57	0
4	VAR1, DS, PS(Awr)	-5258.720	11195.88	0
14	VAR1, PS(Awr)	-5258.858	11196.16	0
3	VAR1, DS	-5255.313	11197.87	0
13	VAR1	-5255.816	11198.87	0
6	DS, PS(Awr)	-5257.887	11220.98	0
16	full	-5250.520	11224.71	0
15	PS(Awr)	-5259.842	11224.89	0
7	DS	-5256.414	11236.49	0
9	VAR1, DS(Shp)	-5444.542	11380.39	0
10	VAR1, DS(Shp), PS(Awr)	-5455.454	11395.85	0
12	DS(Shp), PS(Awr)	-5449.997	11404.27	0
11	DS(Shp)	-5464.192	11445.94	0

TableS6: Summary of the GRT models fitted during the visibility block in Experiment 3 (Local SOA-40)

Model number	Assumptions	Log-likelihood	AIC	AIC weight
2	VAR1, DS(Awr), PS(Awr)	-5684.237	11802.19	1.00
8	DS(Awr), PS(Awr)	-5685.647	11824.59	0
5	DS(Awr)	-5691.990	11850.77	0
1	VAR1, DS(Awr)	-5707.298	11854.75	0
6	DS, PS(Awr)	-5672.559	11979.06	0
15	PS(Awr)	-5680.766	11995.47	0
7	DS	-5672.954	11998.67	0
16	full	-5692.494	12037.75	0
14	VAR1, PS(Awr)	-5716.601	12039.98	0
12	DS(Shp), PS(Awr)	-5798.606	12050.51	0
4	VAR1, DS, PS(Awr)	-5726.764	12060.31	0
3	VAR1, DS	-5739.728	12095.15	0
11	DS(Shp)	-5823.561	12113.91	0
13	VAR1	-5753.681	12123.06	0
9	VAR1, DS(Shp)	-5989.900	12419.96	0
10	VAR1, DS(Shp), PS(Awr)	-6068.080	12569.87	0

TableS7: Summary of the GRT models fitted during the multiple-task block in Experiment 4 (Local SOA-53)

Model number	Assumptions	Log-likelihood	AIC	AIC weight
1	VAR1, DS(Awr)	-5050.170	10540.50	.711
2	VAR1, DS(Awr), PS(Awr)	-5054.299	10542.31	.287
8	DS(Awr), PS(Awr)	-5049.633	10552.56	.002
5	DS(Awr)	-5050.900	10568.59	0
14	VAR1, PS(Awr)	-5025.150	10657.08	0
4	VAR1, DS, PS(Awr)	-5028.511	10663.80	0
13	VAR1	-5024.437	10664.57	0
3	VAR1, DS	-5027.226	10670.14	0
15	PS(Awr)	-5030.794	10695.52	0
6	DS, PS(Awr)	-5035.447	10704.83	0
16	full	-5028.268	10709.30	0
7	DS	-5031.911	10716.58	0
10	VAR1, DS(Shp), PS(Awr)	-5196.403	10826.52	0
9	VAR1, DS(Shp)	-5195.931	10832.02	0
12	DS(Shp), PS(Awr)	-5195.518	10844.33	0
11	DS(Shp)	-5194.222	10855.24	0

TableS8: Summary of the GRT models fitted during the visibility block in Experiment 4 (Local SOA-53)

2. Estimated GRT-wIND models

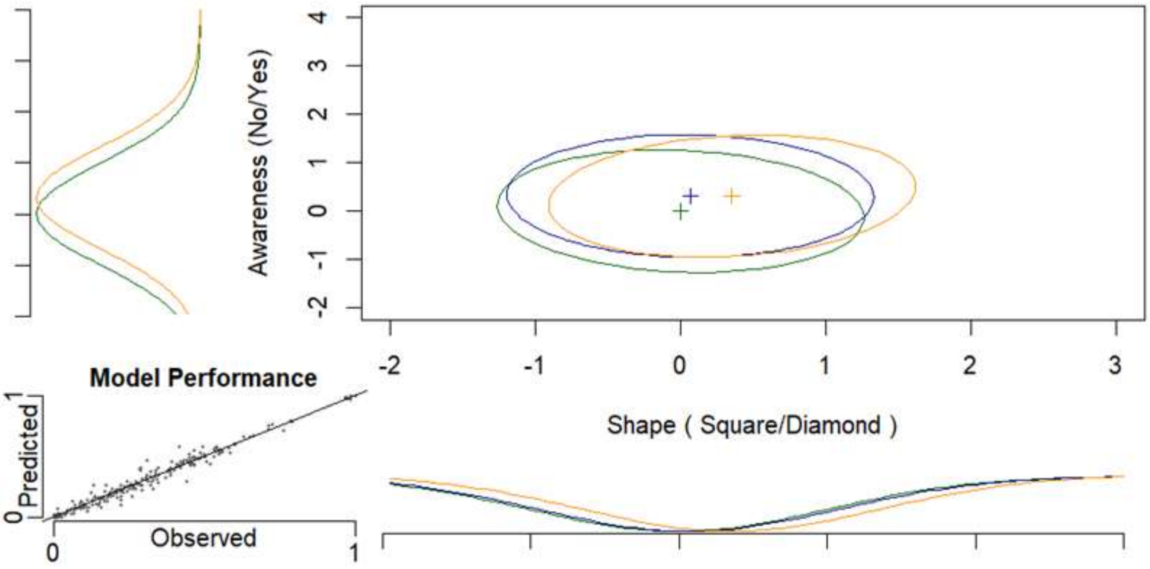

Figure S1: Best-fitting model from the analysis of the discrimination task in the multiple-task block Experiment 1 (Global SOA-40).

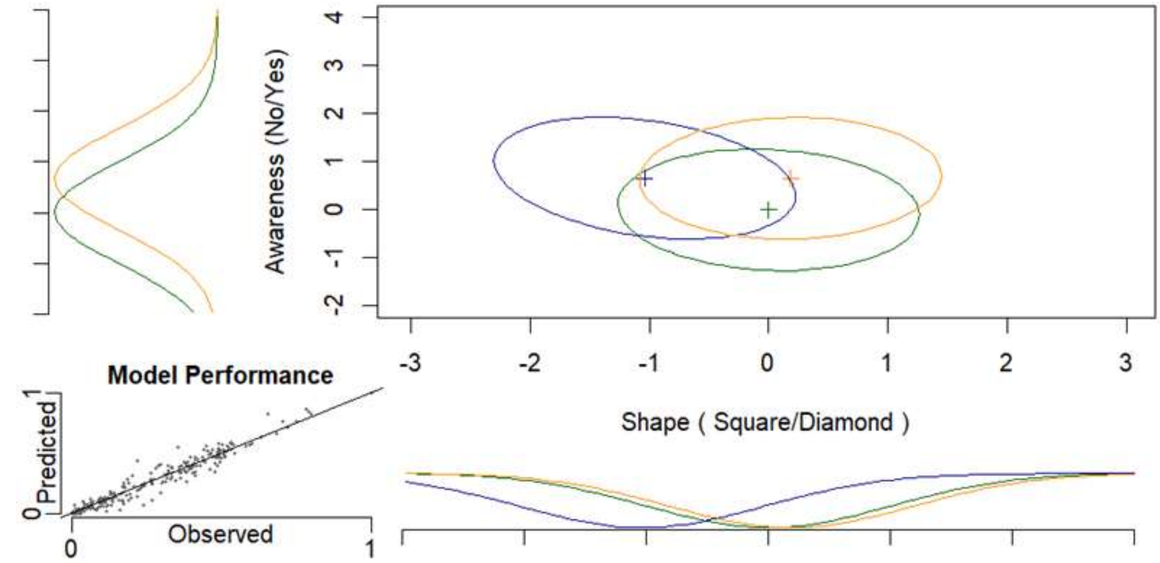

Figure S2: Best-fitting model from the analysis of the discrimination task in the visibility block Experiment 1 (Global SOA-40).

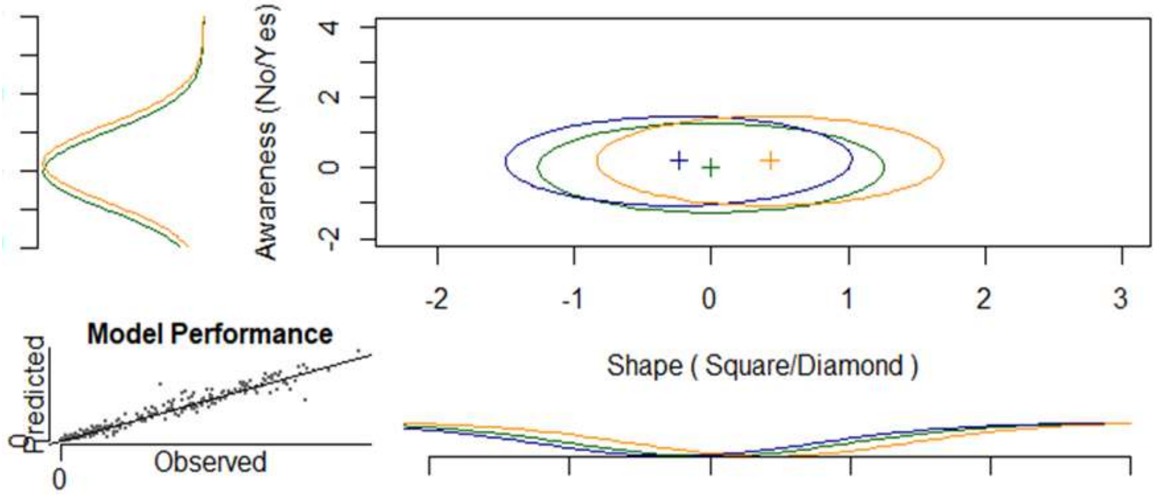

Figure S3: Best-fitting model from the analysis of the discrimination task in the multiple-task block Experiment 1 (Global SOA-53).

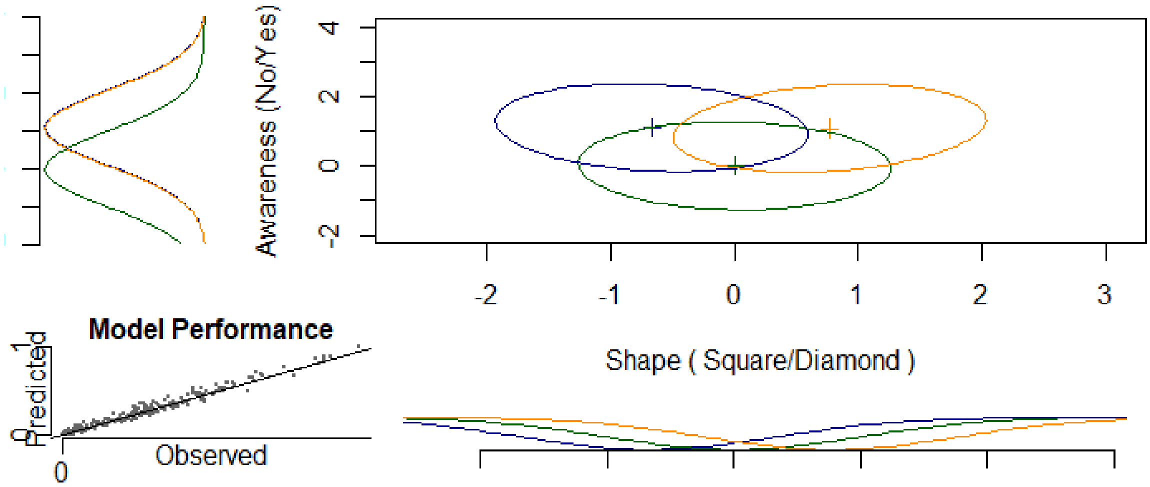

Figure S4: Best-fitting model from the analysis of the discrimination task in the visibility block Experiment 1 (Global SOA-53).

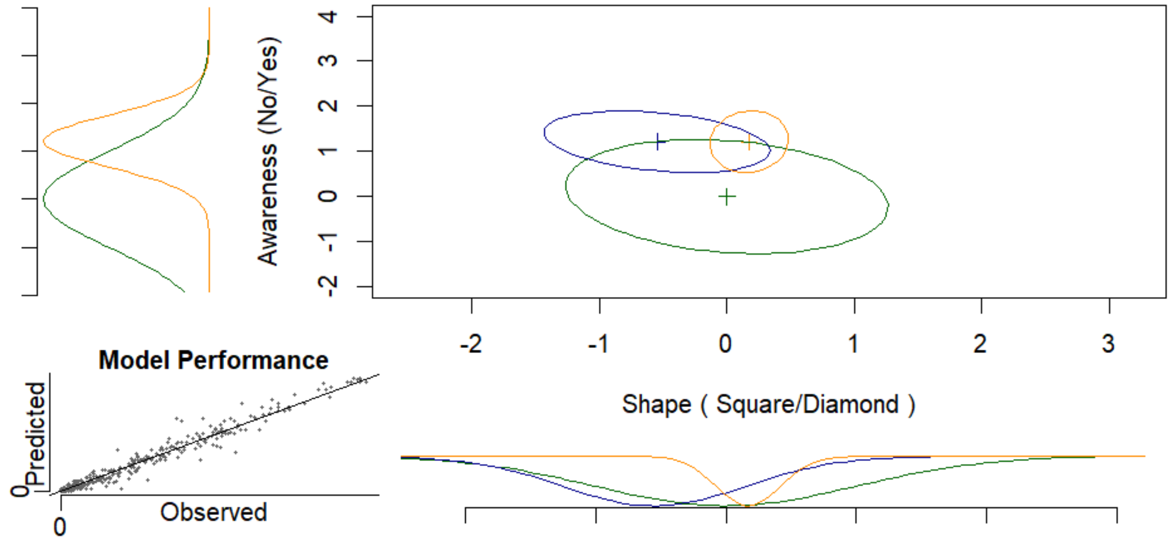

Figure S5: Best-fitting model from the analysis of the discrimination task in the multiple-task block Experiment 1 (Local SOA-40).

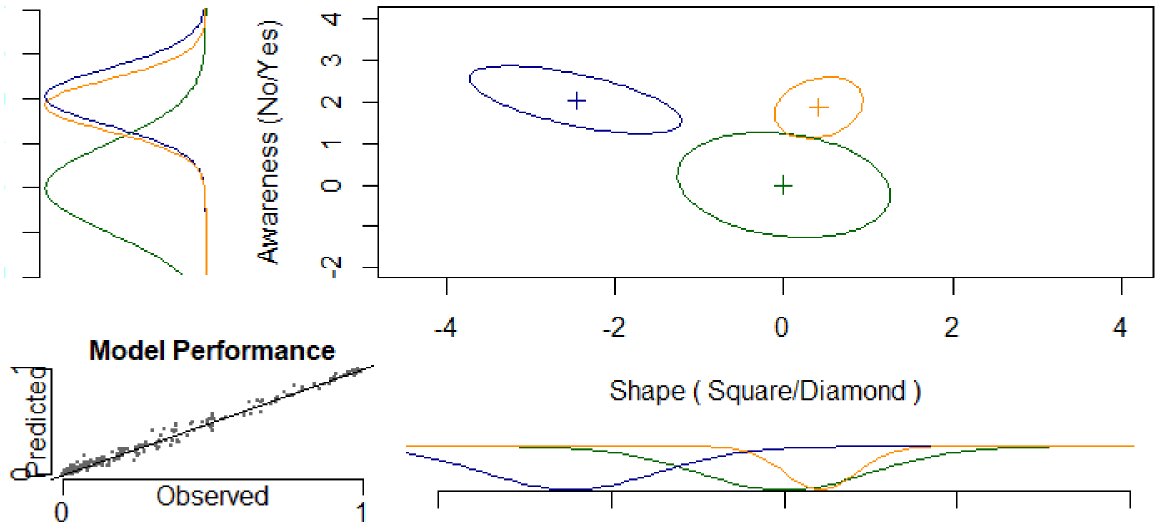

Figure S6: Best-fitting model from the analysis of the discrimination task in the visibility block Experiment 1 (Local SOA-40).

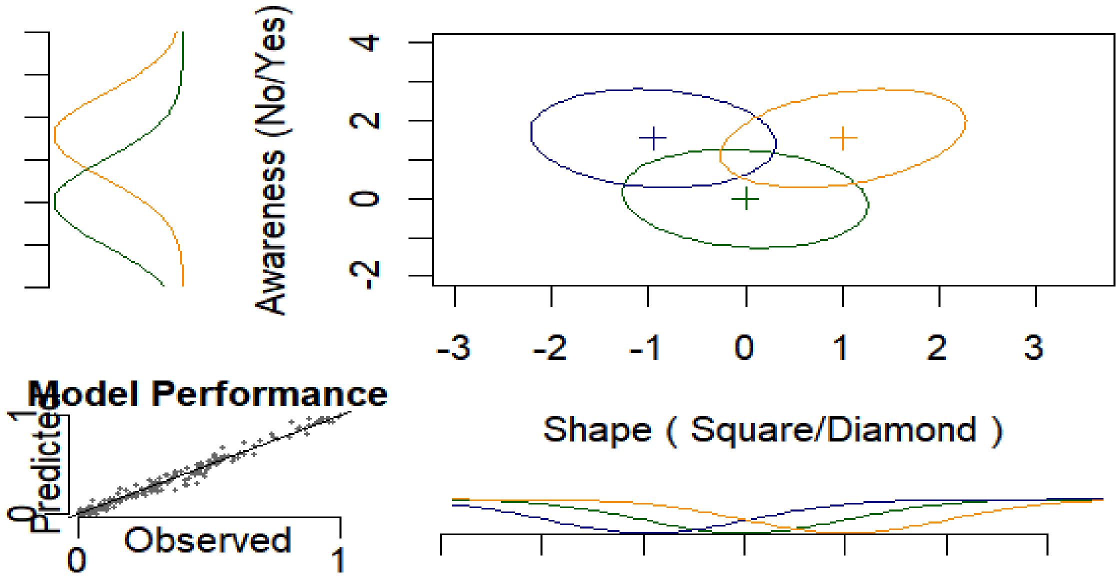

Figure S7: Best-fitting model from the analysis of the discrimination task in the multiple-task block Experiment 1 (Local SOA-53).

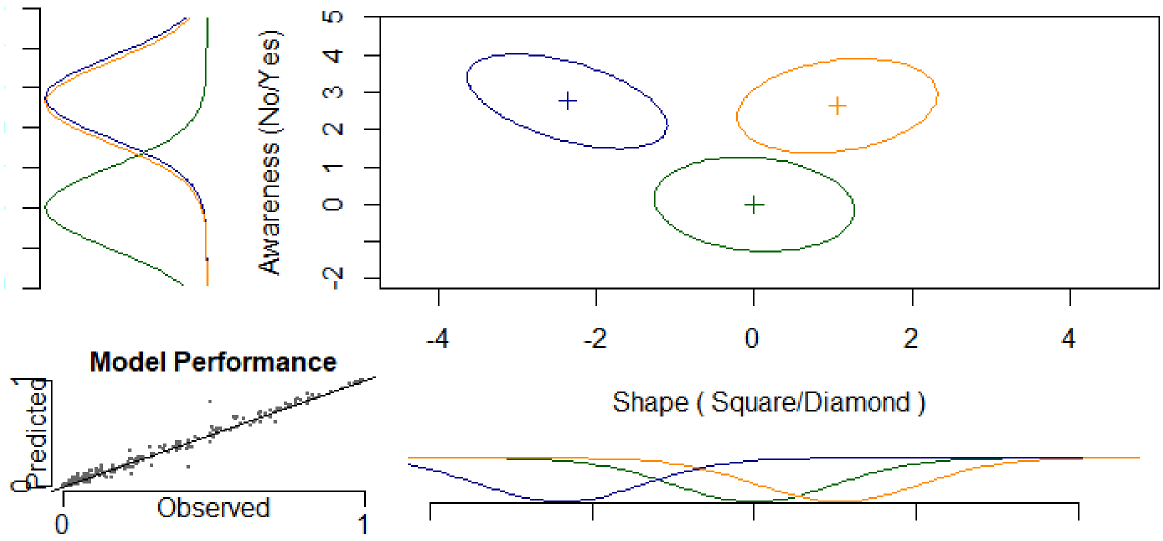

Figure S8: Best-fitting model from the analysis of the discrimination task in the visibility block Experiment 1 (Local SOA-53).

3. Estimated Bayesian generative model correction to Greenwald regression.

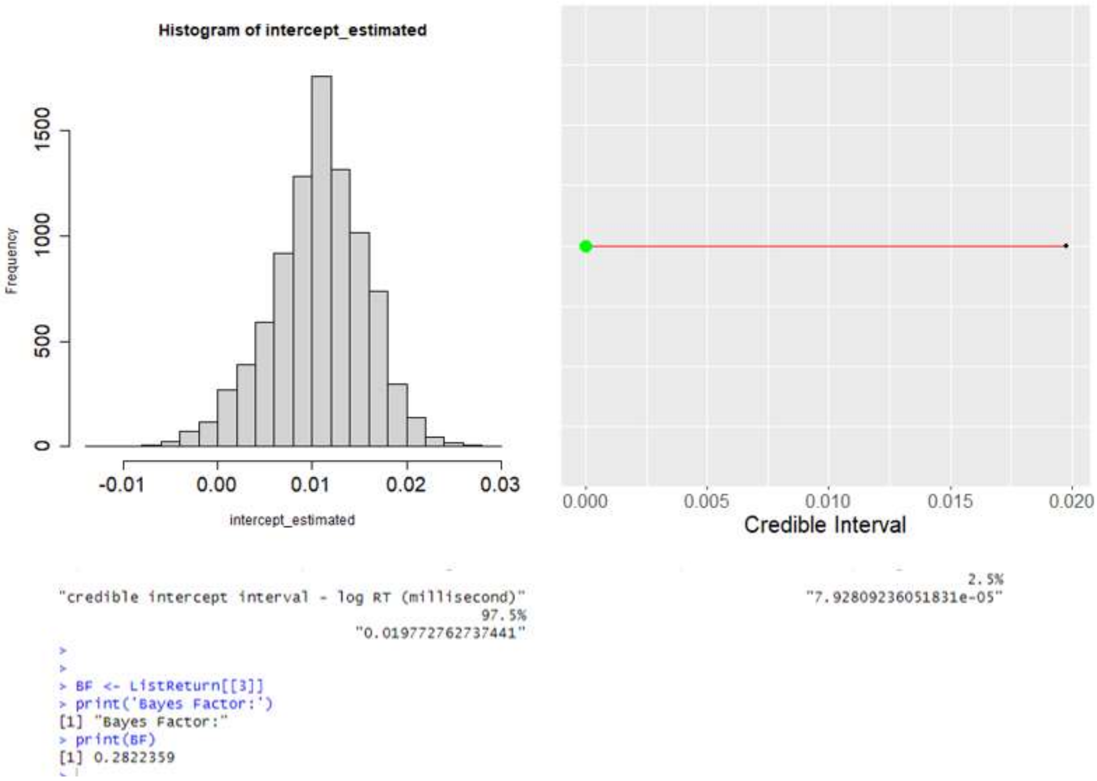

Figure S9: The Output of the Bayesian Model for the data from the single-task block in Experiment 1 (Global SOA-40). (**Upper-left**) Histogram of the posterior estimation of the intercept (in log RT units). (**Upper-right**) Credible interval (2.5, 97.5) for the estimation. (**Bottom**) The values for the credible interval as well as the Bayes factor comparing a null model where the intercept is set to zero (i.e., no nonconscious processing) to a model where it is drawn from wide normal distribution.

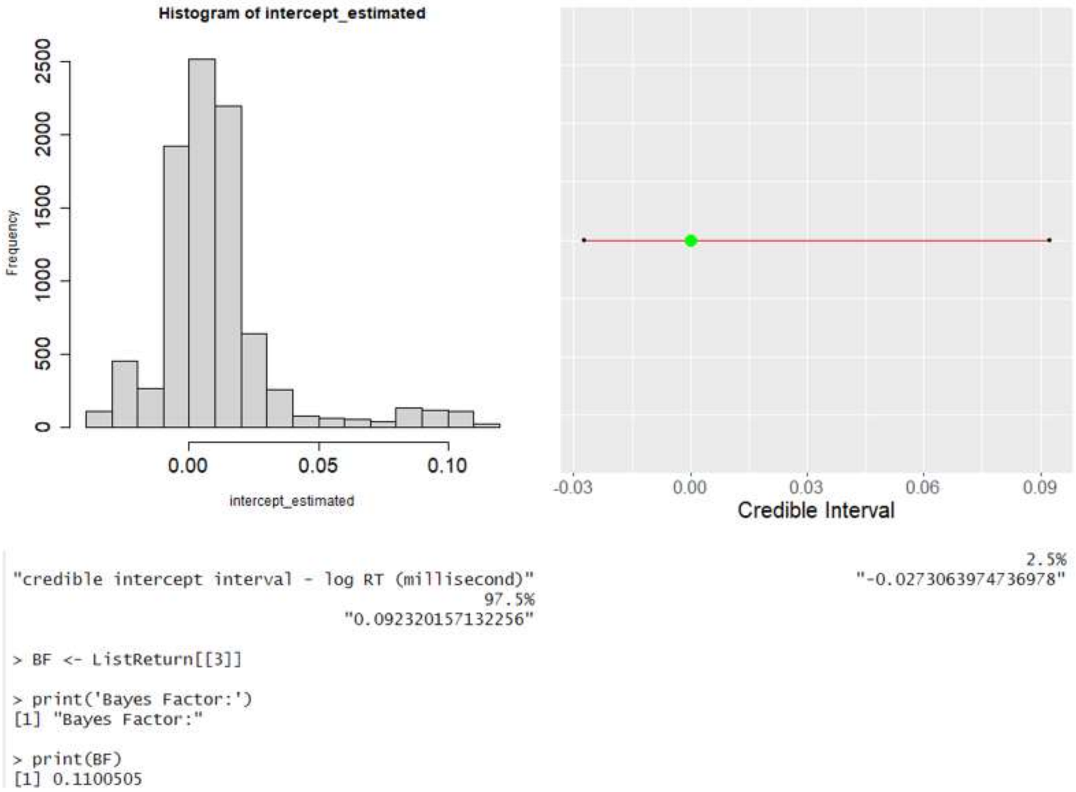

Figure S10: The Output of the Bayesian Model for the data from the multiple-task block in Experiment 1 (Global SOA-40). (**Upper-left**) Histogram of the posterior estimation of the intercept (in log RT units). (**Upper-right**) Credible interval (2.5, 97.5) for the estimation. (**Bottom**) The values for the credible interval as well as the Bayes factor comparing a null model where the intercept is set to zero (i.e., no nonconscious processing) to a model where it is drawn from wide normal distribution.

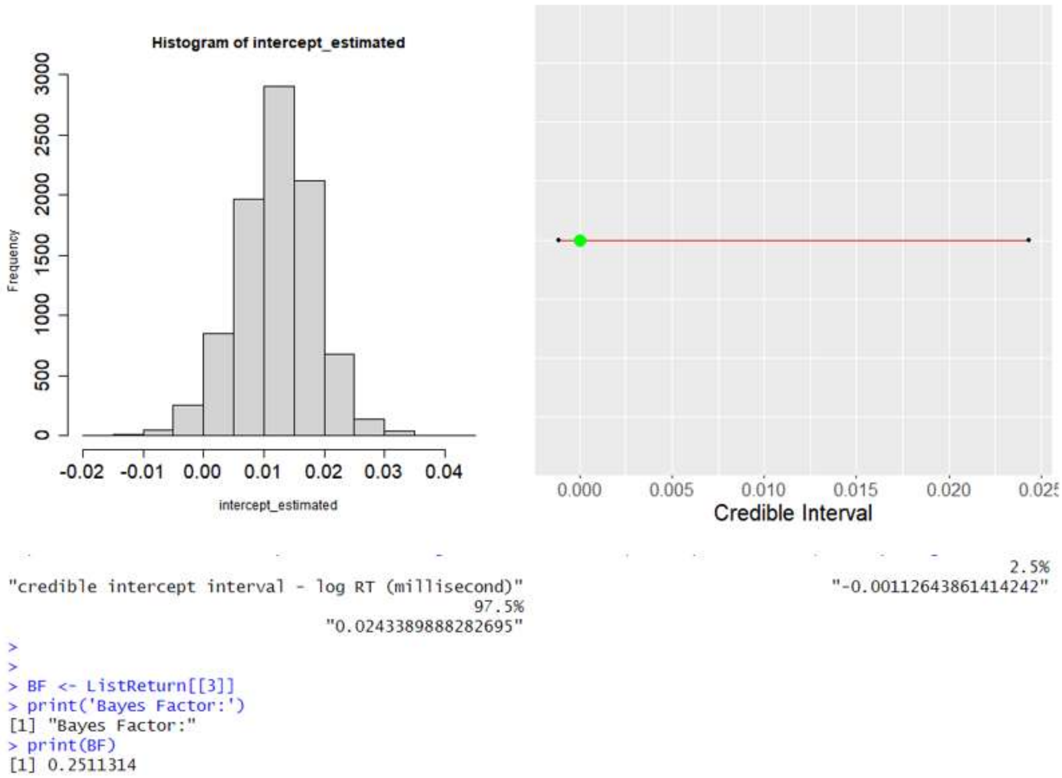

Figure S11: The Output of the Bayesian Model for the data from the single-task block in Experiment 2 (Global SOA-53). (**Upper-left**) Histogram of the posterior estimation of the intercept (in log RT units). (**Upper-right**) Credible interval (2.5, 97.5) for the estimation. (**Bottom**) The values for the credible interval as well as the Bayes factor comparing a null model where the intercept is set to zero (i.e., no nonconscious processing) to a model where it is drawn from wide normal distribution.

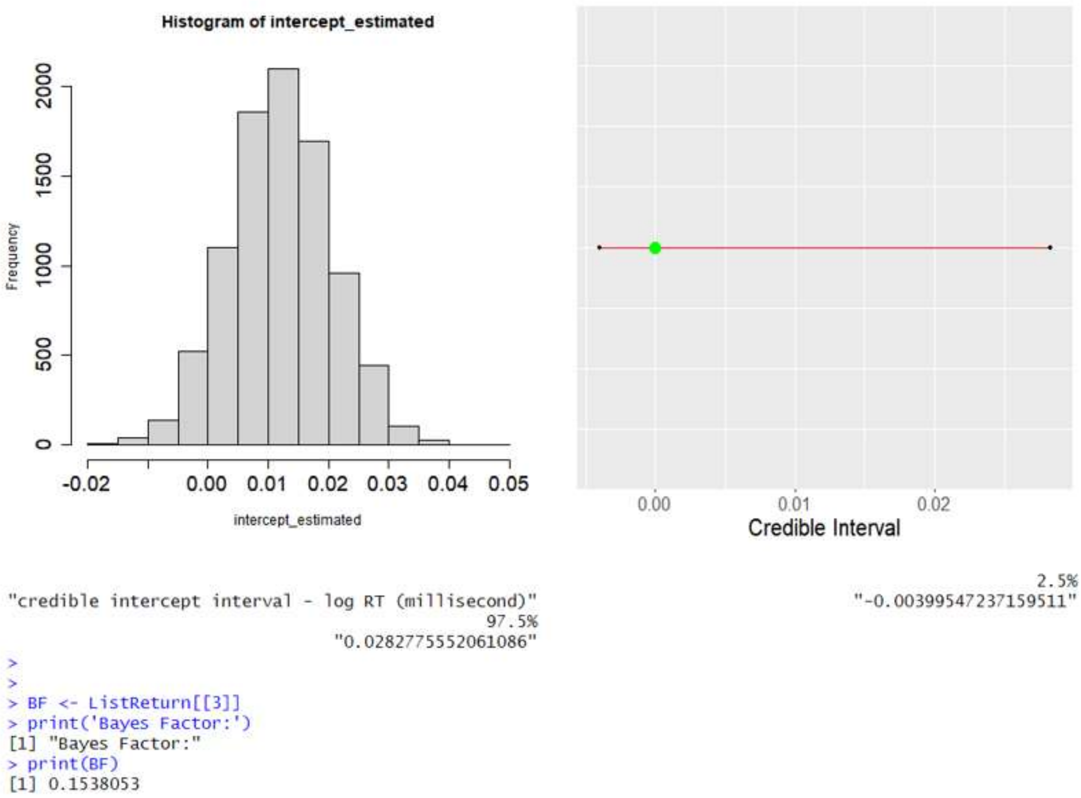

Figure S12: The Output of the Bayesian Model for the data from the multiple-task block in Experiment 2 (Global SOA-53). (**Upper-left**) Histogram of the posterior estimation of the intercept (in log RT units). (**Upper-right**) Credible interval (2.5, 97.5) for the estimation. (**Bottom**) The values for the credible interval as well as the Bayes factor comparing a null model where the intercept is set to zero (i.e., no nonconscious processing) to a model where it is drawn from wide normal distribution.

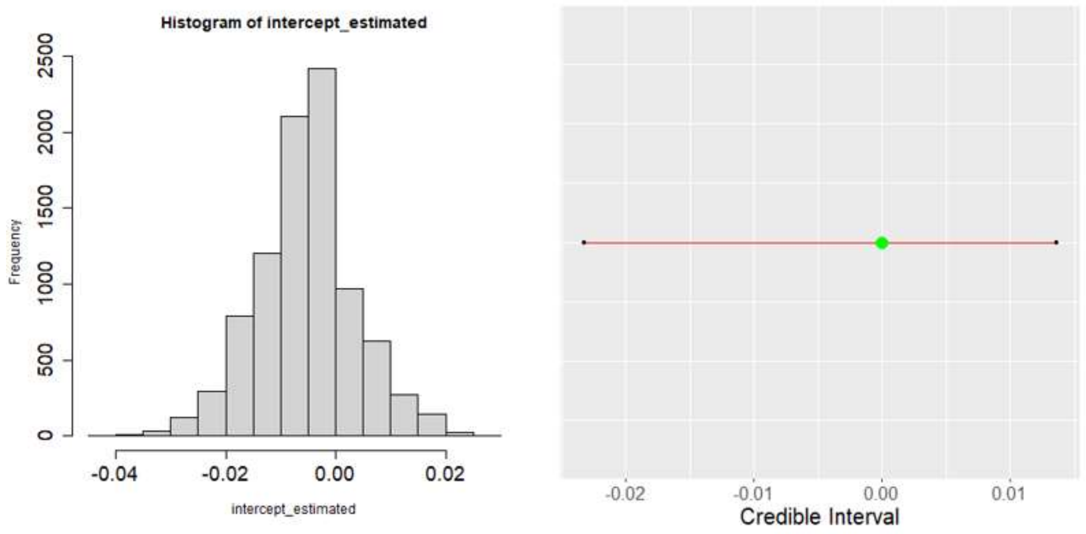


```
"credible intercept interval - log RT (millisecond)"                                    2.5%
                                                                       "-0.0232338681353143"
                                                 97.5%
                                 "0.0136129903487976"

> BF <- ListReturn[[3]]

> print('Bayes Factor:')
[1] "Bayes Factor:"

> print(BF)
[1] 0.08582489
```

Figure S13: The Output of the Bayesian Model for the data from the single-task block in Experiment 3 (Local SOA-40). (**Upper-left**) Histogram of the posterior estimation of the intercept (in log RT units). (**Upper-right**) Credible interval (2.5, 97.5) for the estimation. (**Bottom**) The values for the credible interval as well as the Bayes factor comparing a null model where the intercept is set to zero (i.e., no nonconscious processing) to a model where it is drawn from wide normal distribution.

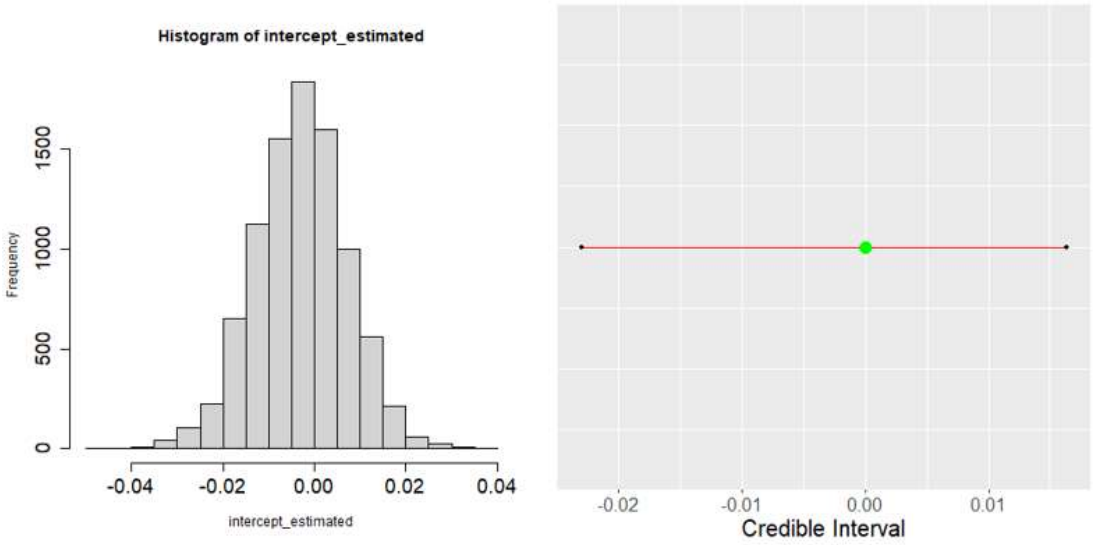


```
"credible intercept interval - log RT (millisecond)"                          2.5%
                                                 97.5%     "-0.0212744814628066"
                                 "0.017481456189955"

> BF <- ListReturn[[3]]

> print('Bayes Factor:')
[1] "Bayes Factor:"

> print(BF)
[1] 0.07242006
```

Figure S14: The Output of the Bayesian Model for the data from the multiple-task block in Experiment 3 (Local SOA-40). (**Upper-left**) Histogram of the posterior estimation of the intercept (in log RT units). (**Upper-right**) Credible interval (2.5, 97.5) for the estimation. (**Bottom**) The values for the credible interval as well as the Bayes factor comparing a null model where the intercept is set to zero (i.e., no nonconscious processing) to a model where it is drawn from wide normal distribution.

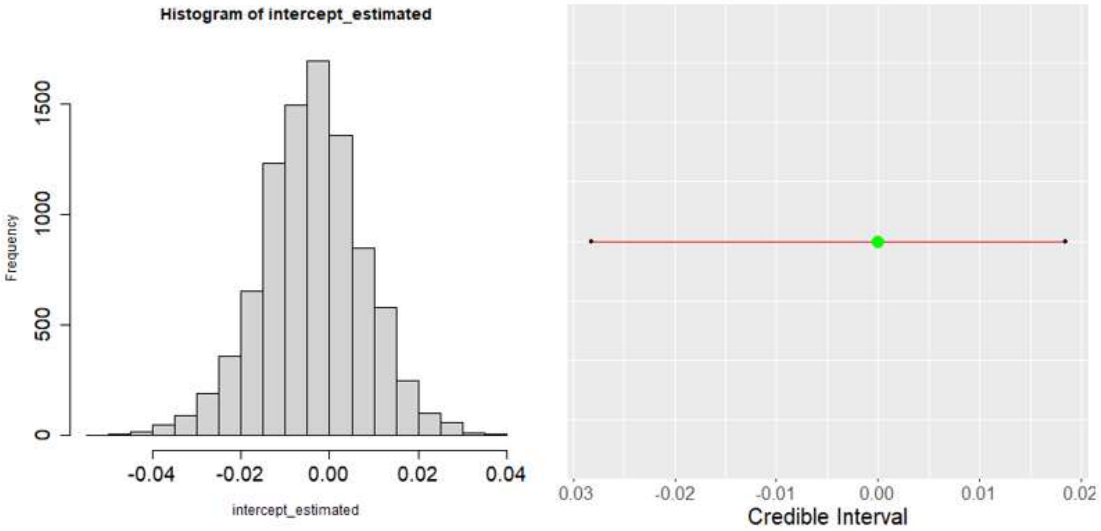


```
                                                        2.5%
"credible intercept interval - log RT (millisecond)"   "-0.0282228801554202"
                                                        97.5%
                                                        "0.0184395560358684"

> BF <- ListReturn[[3]]

> print('Bayes Factor:')
[1] "Bayes Factor:"

> print(BF)
[1] 0.07600639
```

Figure S15: The Output of the Bayesian Model for the data from the single-task block in Experiment 4 (Local SOA-53). (**Upper-left**) Histogram of the posterior estimation of the intercept (in log RT units). (**Upper-right**) Credible interval (2.5, 97.5) for the estimation. (**Bottom**) The values for the credible interval as well as the Bayes factor comparing a null model where the intercept is set to zero (i.e., no nonconscious processing) to a model where it is drawn from wide normal distribution.

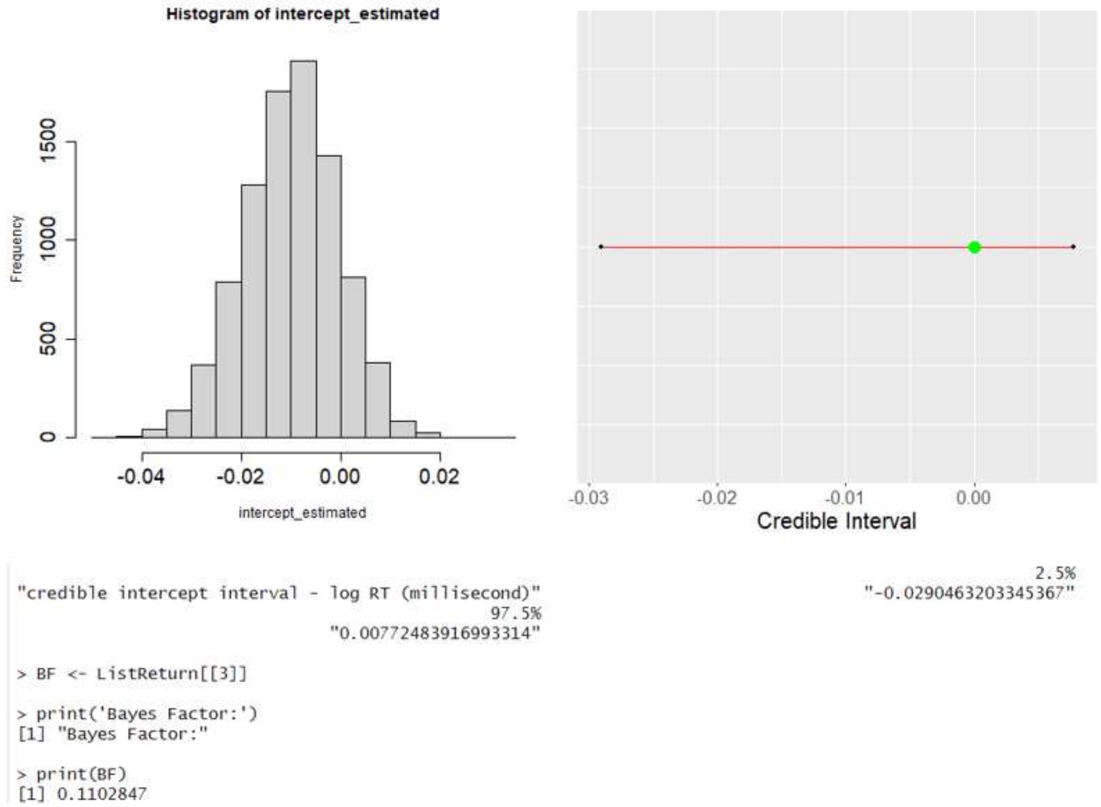

Figure S16: The Output of the Bayesian Model for the data from the multiple-task block in Experiment 4 (Local SOA-53). (**Upper-left**) Histogram of the posterior estimation of the intercept (in log RT units). (**Upper-right**) Credible interval (2.5, 97.5) for the estimation. (**Bottom**) The values for the credible interval as well as the Bayes factor comparing a null model where the intercept is set to zero (i.e., no nonconscious processing) to a model where it is drawn from wide normal distribution.

4. RT: Single-task vs. multiple-task comparison

To compare RTs between the single and multiple-task priming blocks, individual mean RTs from both priming blocks in each experiment entered a 2 x 2 Bayesian repeated-measures ANOVA, with Block (single-task, multiple-task) and Congruency (congruent, incongruent) as within-subject factors.

4.1. Experiment 1 (Global SOA40)

Model comparison showed that a model with Block and Congruency as main factors explained the data 1.71 times better than a model with only Block as main factor (Block and Congruency: BF_{10} = 2399.28, error % = 3.241; Block: BF_{10} = 1406.58, error % = 3.930; both against the null model without factors). Averaging the conclusions from each candidate model weighted by that model's posterior plausibility (i.e., Bayesian Model Averaging) showed strong evidence in favor of block differences on RTs (Block: BF_{incl} = 1083.492), congruent with the faster RTs found in the single-task block. The analysis also showed anecdotal evidence favoring a congruency effect (Congruency: BF_{incl} = 1.49), and anecdotal evidence against a Block x

Congruency interaction (Block x Congruency: $BF_{incl} = 0.78$) (single-task: Congruent: $M = 518$ ms, Incongruent: $M = 527$ ms; dual-task: Congruent: $M = 780$ ms, Incongruent: $M = 786$ ms; see **Table 2** and **Figure 3** in the main text), suggesting no interactive effects between Block and Congruency nor a main effect of Congruency.

4.2. Experiment 2 (Global SOA53)

Mean RTs from both primming blocks entered a 2 x 2 Bayesian repeated-measures ANOVA, with Block (single-task, multiple-task) and Congruency (congruent, incongruent) as within-subject factors. Model comparison showed that a model with Block as the main factor explained the data 1.09 times better than a model with Block and Congruency as main factors (Block: BF10 = 7248.563, error % = 3.716; Block and Conguency: BF10 = 6646.826, error % = 3.714; both against the null model without factors). Averaging the conclusions from each candidate model weighted by that model's posterior plausibility showed strong evidence in favor of block differences on RTs (Block: BFincl = 6115.94). congruent with the faster RTs in the single-task block (single-task: Congruent: M = 548 ms, Incongruent: M = 554 ms; dual-task: Congruent: M = 859 ms, Incongruent: M = 870 ms; see **Table 2** and **Figure 3** in the main text). Anecdotal evidence against a Congruency effect and Block x Congruency interaction was also found (Congruency: BFincl = 0.925; Task x Congruency: BFincl = 0.981) suggesting neither a main effect of Congruency nor an interactive effect between Block and Congruency.

4.3. Experiment 3 (Local SOA40)

Individual mean RTs from both primming blocks entered a 2 x 2 Bayesian repeated-measures ANOVA, with Block (single-task, multiple-task) and Congruency (congruent, incongruent) as within-subject factors. Model comparison showed that a model with Block and Congruency as main factors and the interaction between Block and Congruency explained the data 2.97 times better than a model with only Block as main factor (Block, Congruency and Block x Congruency: $BF_{10} = 332020.71$, error % = 4.041; Block: $BF_{10} = 111794.83$, error % = 4.560; both against the null model without factors). Averaging the conclusions from each candidate model weighted by that model's posterior plausibility showed strong evidence in favor of block differences on RTs (Block: $BF_{incl} = 182187.52$), congruent with the faster RTs found in the single-task block. The analysis also showed moderate evidence favoring a Block x Congruency effect ($BF_{incl} = 6.234$), indicating that congruency effects appeared only in the multiple-task block (single-task: Congruent: $M = 520$ ms, Incongruent: $M = 519$ ms; dual-task: Congruent: $M = 858$ ms, Incongruent: $M = 887$ ms; see **Table 2** and **Figure 3** in the main text). Anecdotal evidence favoring a Congruency main effect was also found (Congruency: $BF_{incl} = 2.584$).

4.4. Experiment 4 (Local SOA53)

Individual mean RTs from both primming blocks entered a 2 x 2 Bayesian repeated-measures ANOVA, with Block (single-task, multiple-task) and Congruency (congruent, incongruent) as within-subject factors. Model comparison showed that a model including Block and Congruency as main factors and the interaction between Block and Congruency explained the data 2.18 times better than a model with only Block as main

factor (Block, Congruency and Block x Congruency: BF_{10} = 15218.14, error % = 5.133; Block: BF_{10} = 6995.74, error % = 1.898; both against the null model without factors). Averaging the conclusions from each candidate model weighted by that model's posterior plausibility showed strong evidence in favor of block differences on RTs (Block: BF_{incl} = 11027.59), congruent with the faster RTs in the single-task block. The analysis also showed moderate evidence favoring a Block x Congruency effect (BF_{incl} = 5.048), indicating that differences in congruency appeared in the multiple-task block and not in the single-task block: (single-task: Congruent: *M* = 569 ms, Incongruent: *M* = 565 ms; dual-task: Congruent: *M* = 951 ms, Incongruent: *M* = 982 ms; see **Table 2** and **Figure 3** in the main text). Anecdotal evidence favoring a Congruency main effect was also found (Congruency: BF_{incl} = 1.932).

5. Correlations matrices between the different sensitivity measures (d').

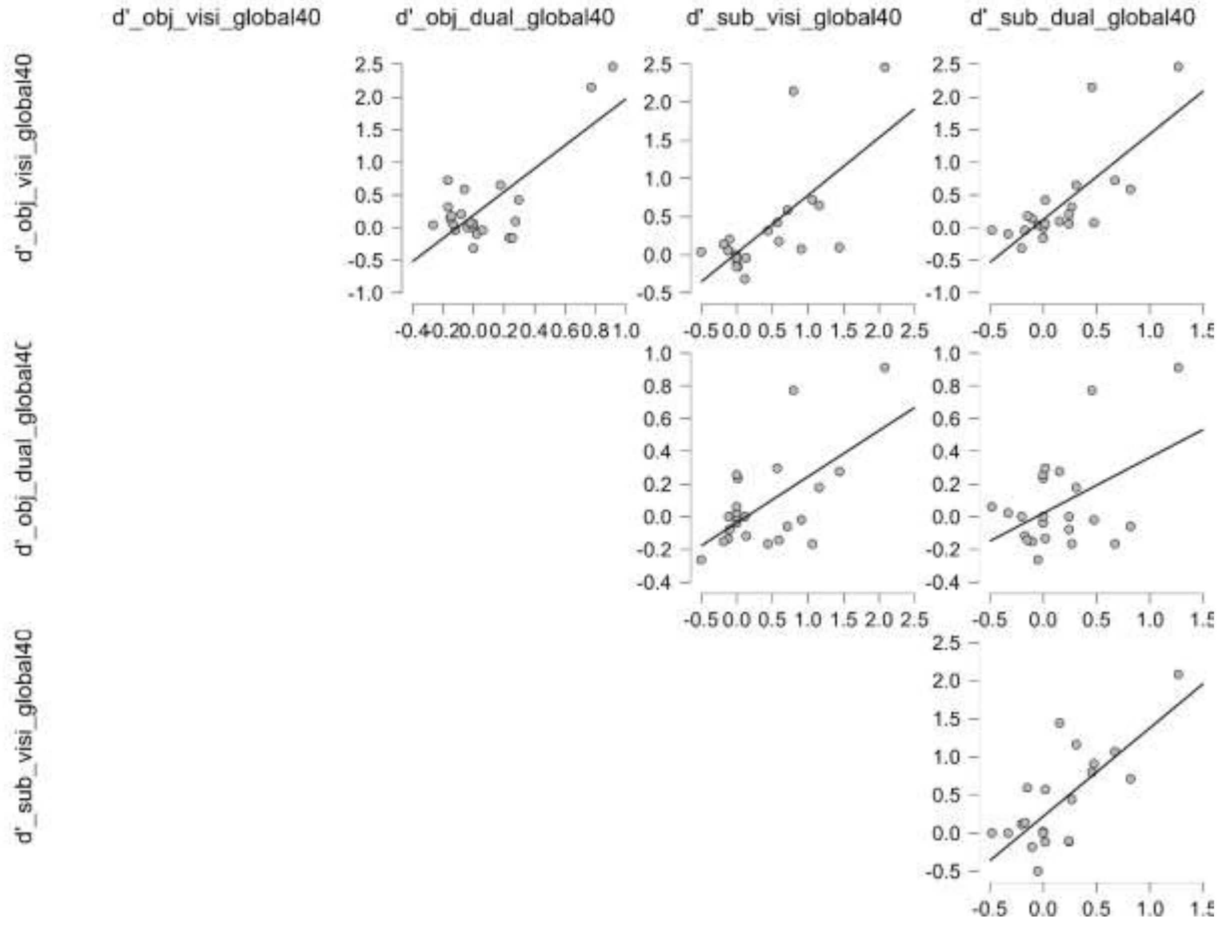

Figure S17: Correlation matrix between d'_{obj} and d'_{subj} collected during the multiple-task and visibility blocks in Experiment 1 (Global SOA-40).

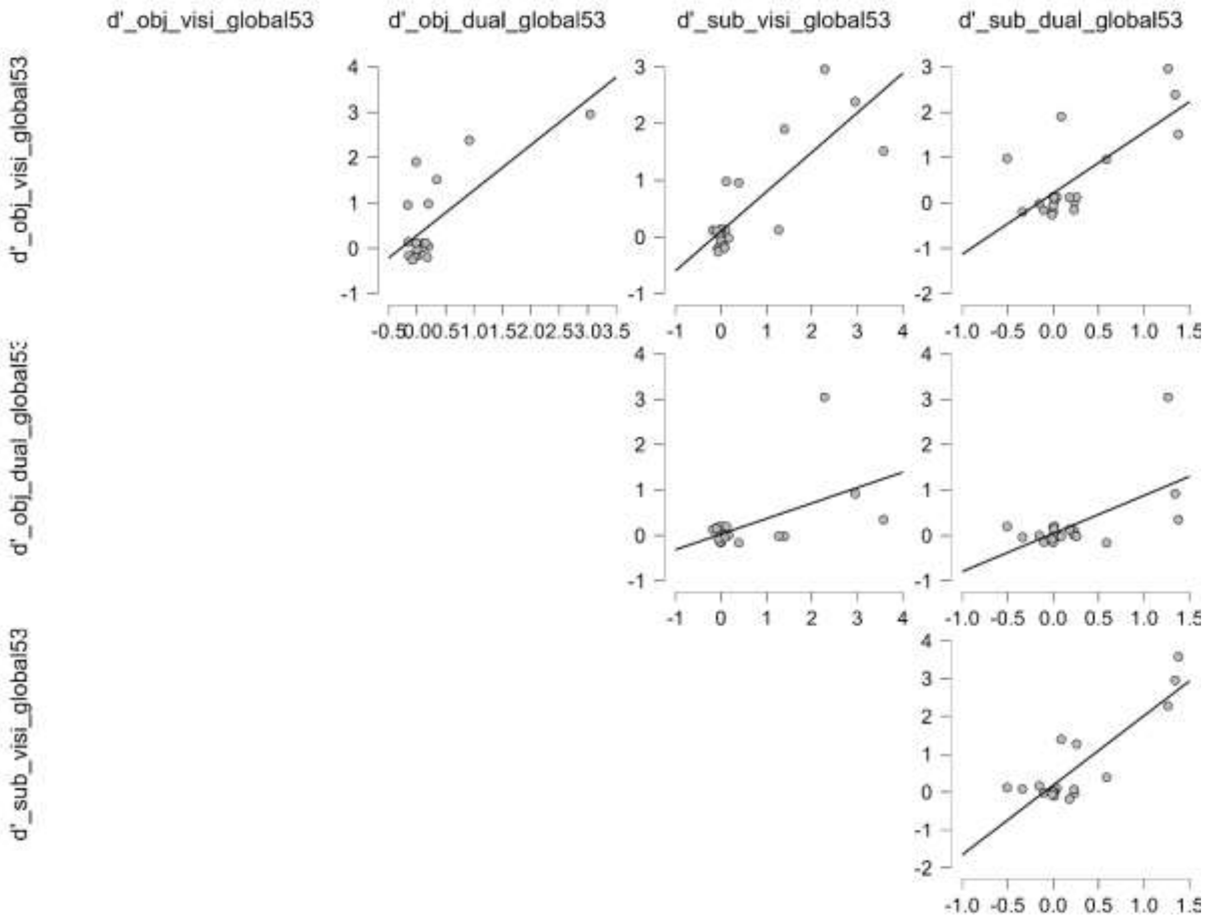

Figure S17: Correlation matrix between d'$_{obj}$ and d'$_{subj}$ collected during the multiple-task and visibility blocks in Experiment 2 (Global SOA-53).

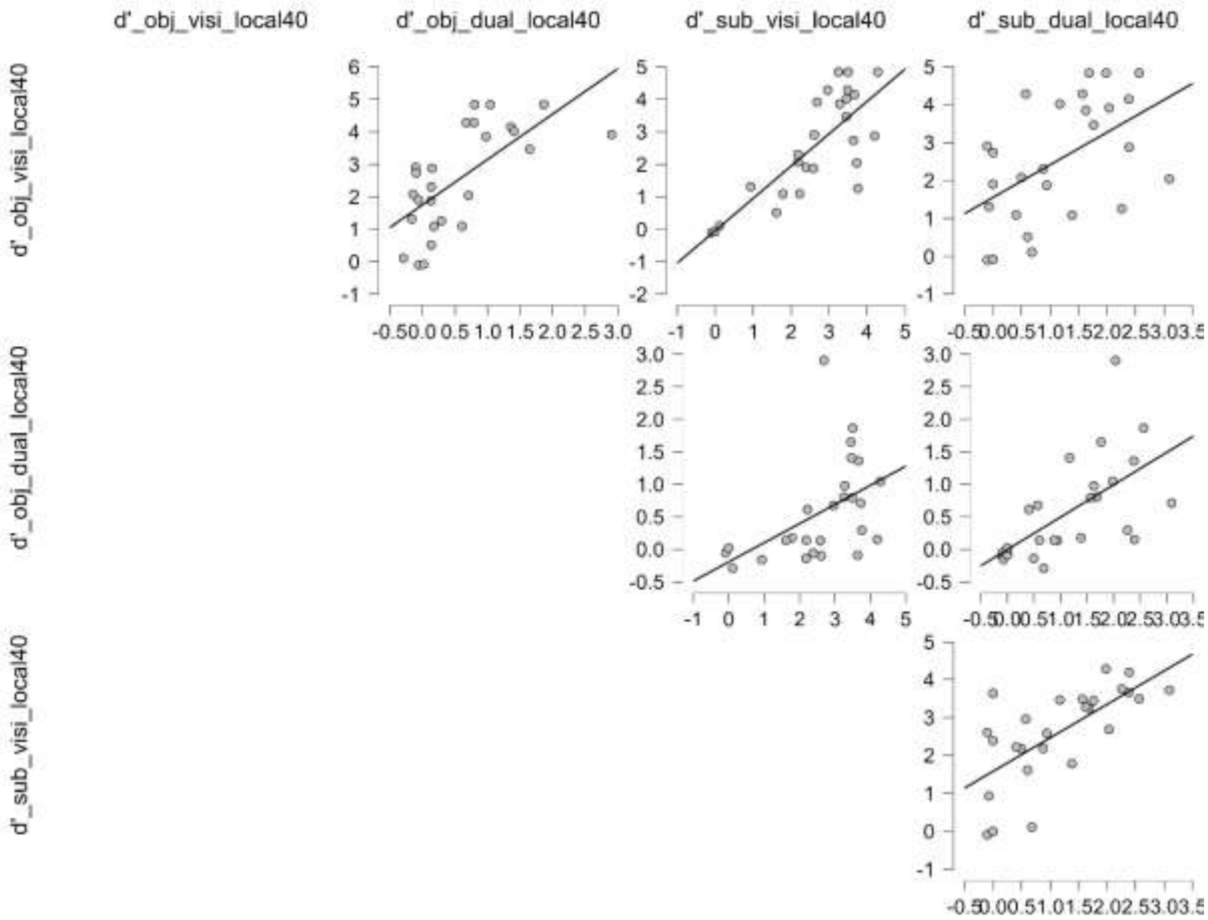

Figure S17: Correlation matrix between d'$_{obj}$ and d'$_{subj}$ collected during the multiple-task and visibility blocks in Experiment 3 (Local SOA-40).

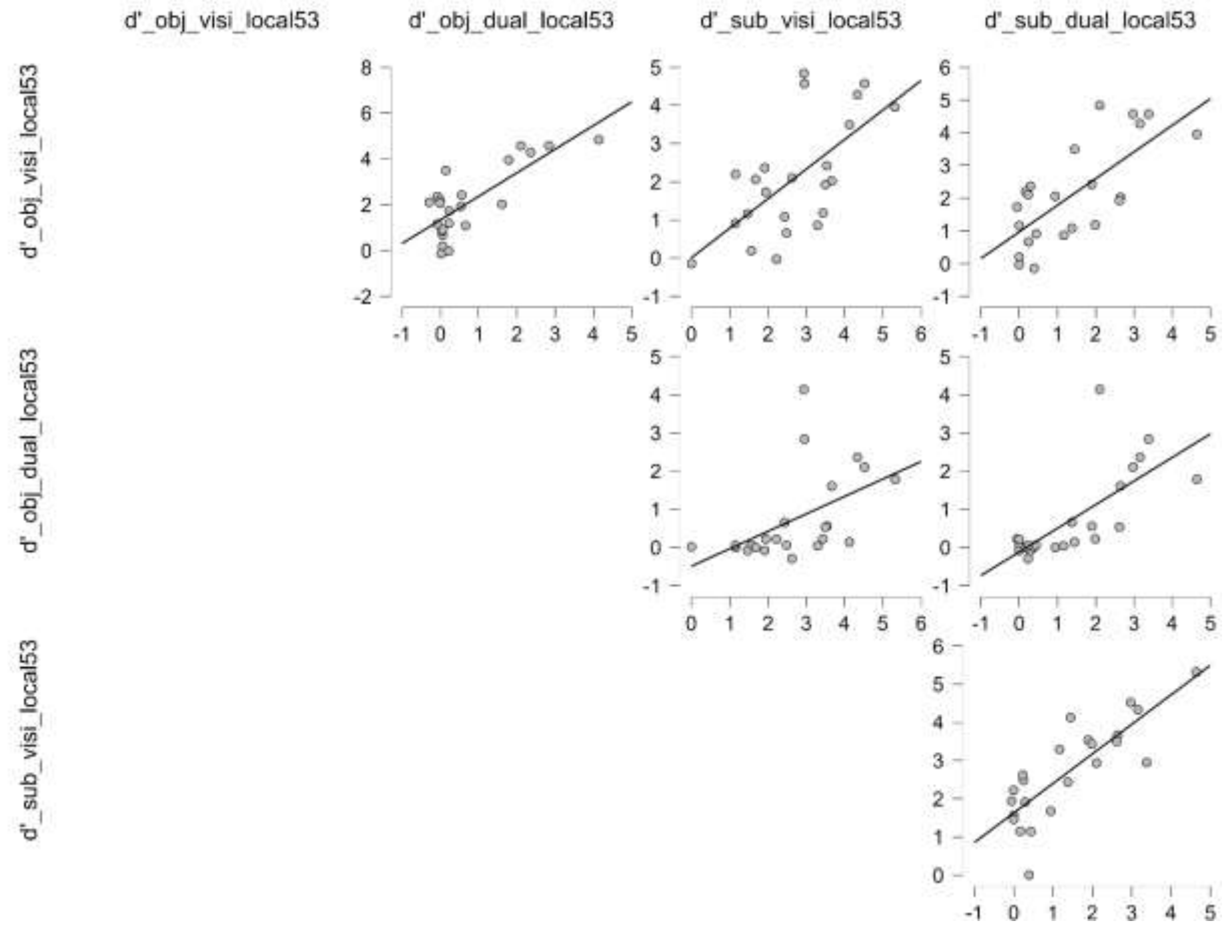

Figure S17: Correlation matrix between d'$_{obj}$ and d'$_{subj}$ collected during the multiple-task and visibility blocks in Experiment 4 (Local SOA-53).
